# The geography of evolutionary divergence in the highly endemic avifauna from the Sierra Madre del Sur, Mexico

**DOI:** 10.1186/s12862-019-1564-3

**Published:** 2019-12-30

**Authors:** Alberto Rocha-Méndez, Luis A. Sánchez-González, Clementina González, Adolfo G. Navarro-Sigüenza

**Affiliations:** 1 Museo de Zoología, Facultad de Ciencias, Universidad Nacional Autónoma de México, Apartado Postal 70-399, 04510 Mexico City, Mexico; 2 Posgrado en Ciencias Biológicas, Universidad Nacional Autónoma de Mexico, Mexico City, Mexico; 3 Instituto de Investigaciones sobre los Recursos Naturales, Universidad Michoacana de San Nicolás de Hidalgo, Morelia, Mexico

**Keywords:** Approximate Bayesian computation, Coalescent, Comparative phylogeography, Mesoamerica, Mexico, Montane forest, mtDNA, Pleistocene, Sierra Madre del Sur

## Abstract

**Background:**

Mesoamerica is a remarkable region with a high geological and ecological complexity. Within northern Mesoamerica, the biotic province of the Sierra Madre del Sur (SMS) in southwestern Mexico harbors exceptionally high avian endemism and diversity. Herein, we searched for spatially and temporally concordant phylogeographic patterns, in four bird genera from three distinct avian orders co-distributed across Mesoamerica and investigated their causes through hypothesis testing regarding historical processes. Selected species include endemic and differentiated populations across the montane forests of Mesoamerica, and particularly within the SMS.

**Results:**

We gathered mitochondrial DNA sequences for at least one locus from 177 individuals across all species. We assessed genetic structure, demographic history, and defined a framework for the coalescent simulations used in biogeographic hypothesis testing temporal and spatial co-variance. Our analyses suggested shared phylogeographic breaks in areas corresponding to the SMS populations, and between the main montane systems in Mesoamerica, with the Central Valley of Oaxaca and the Nicaragua Depression being the most frequently shared breaks among analyzed taxa. Nevertheless, dating analyses and divergence patterns observed were consistent with the hypothesis of broad vicariance across Mesoamerica derived from mechanisms operating at distinct times across taxa in the SMS.

**Conclusions:**

Our study provides a framework for understanding the evolutionary origins and historical factors enhancing speciation in well-defined regions within Mesoamerica, indicating that the evolutionary history of extant biota inhabiting montane forests is complex and often idiosyncratic.

## Background

Comparison of phylogeographic patterns across co-distributed species provides valuable insights on the probable congruence of processes that may have driven intraspecific diversification in particular regions [1–4]. Several phylogeographic studies of individual bird species have shown that genetic structure is frequently associated to discontinuous ranges and geographical barriers that restrict admixture between populations, leading to genetic differentiation (see [5–8]) which may have proceeded independently or jointly with the divergence in morphological, behavioral or ecological traits (see [9–13]). However, a relatively lesser effort has been dedicated to provide robust biogeographic hypotheses seeking to explain shared distributional patterns, even when it may be expected that species sharing geographic areas should show congruent spatio-temporal patterns of differentiation [1, 14–19].

Situated at the northernmost Neotropics, Mesoamerica possess one of the highest levels of endemism and species diversity, but also one of the most globally threatened biota and high rates of deforestation [20, 21]. The complex geological history of this region, as well as the cyclic changes in vegetation and climate, pose it as a challenging area for biogeographic and evolutionary studies; in addition, constant orogenic processes have promoted a highly broken topography characterized by highland isolated patches of humid montane forest between 600 and 3000 m, which includes both humid pine-oak forest and cloud forest [22–24]. This mosaic-like landscape has been associated to centers of diversification along elevational gradients, in which both a high species richness and endemism have evolved for the last 2 Myr, likely as a result of both environmental and geological complexity, as well as Pleistocene climatic fluctuations [6, 15, 18, 22, 25–29], which is supported by relatively recent intraspecific differentiation processes in several groups of organisms (see [27, 30]), therefore explaining the existence of numerous endemic species in different taxonomic groups, including birds [31–35].

**Fig. 1:**
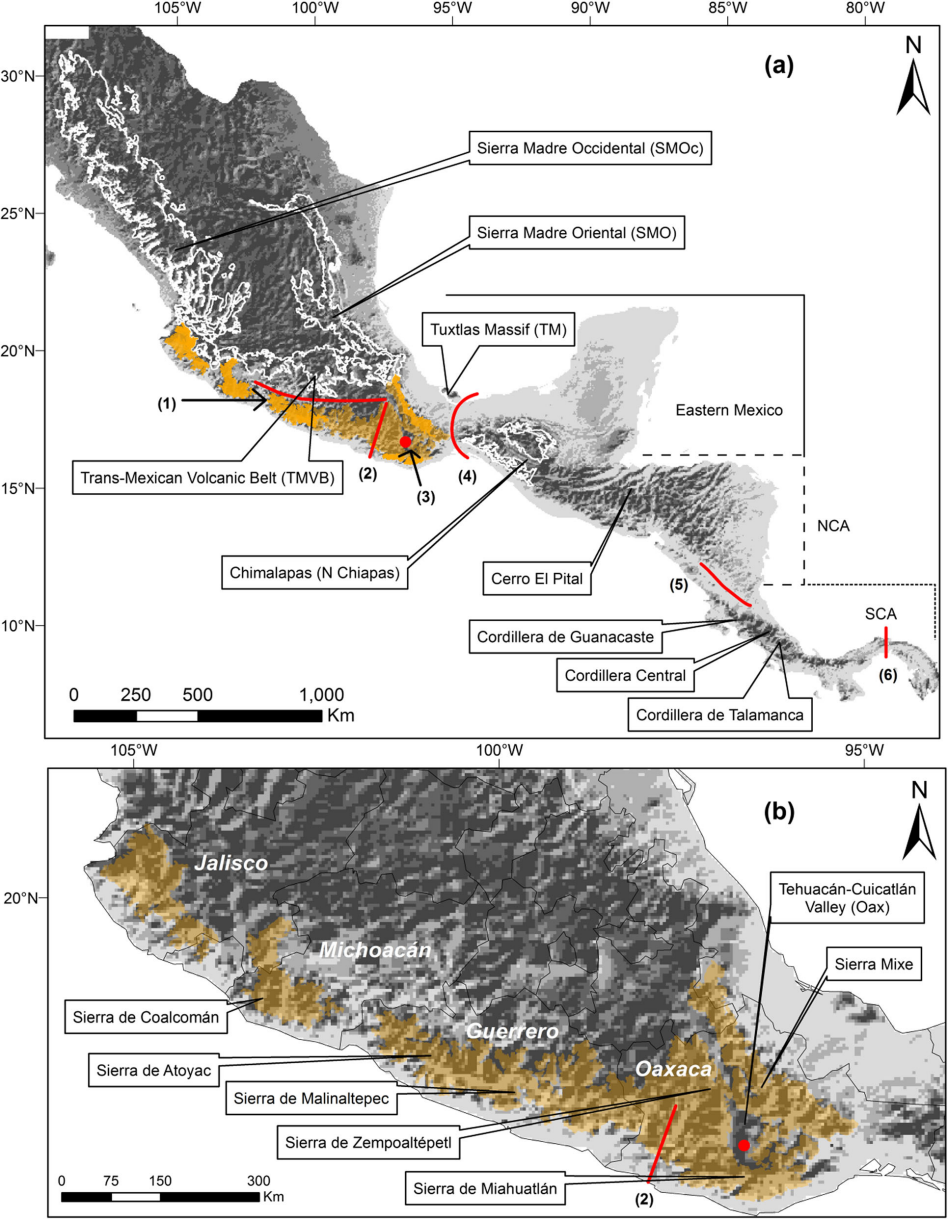
**a** Map of Mesoamerica showing the distribution of montane regions for which genetic data were analyzed in the present study, and summary of the main geographic groups in which they were divided; based on [37] and *Natural Earth* data (http://naturalearthdata.com). Highlighted in orange is the SMS region and main geographical breaks are shown with red lines. Evaluated barriers in this study are numbered as follows: 1) Balsas Depression, 2) Río Verde drainage, 3) Oaxaca’s Central Valley, 4) Isthmus of Tehuantepec, 5) Nicaragua Depression, and 6) Panama Channel Fault Zone (PCFZ). **b** Sierra Madre del Sur mountain range (SMS, ca. 56,729 km^2^) highlighted in orange, showing the broken topography and main mountain peaks. The red dot depicts Oaxaca’s Central Valley, whereas the red line depicts the Río Verde drainage

The Sierra Madre del Sur (SMS) biogeographic province is included within the Mesoamerican highlands, in the Mexican Transition Zone (MTZ, [36]). This isolated mountain range is mostly surrounded by dry lowlands of the Isthmus of Tehuantepec (IT) to the east, the Pacific slope to the south and west, the Balsas Depression and the central Trans-Mexican Volcanic Belt to the north (Fig. 1 [38–41];). The SMS with an overall extension of ca. 56,729 km^2^, is located along the Mexican Pacific slope (Jalisco to Oaxaca, 16–18°N, 95–102°W) [42]. The region hosts 622 avian species, 29 (4.6%) of which are classified as semi-endemic (species breeding elsewhere but for which the entire species’ population spends the wintering season within the political limits of Mexico [43]), 15 (2.4%) as quasi-endemic (species whose geographic range extends no more than 35,000 km^2^ to a neighboring country due to habitat continuity, [43]), and 54 (8.6%) are endemic to Mexico; in addition, 134 (21.5%) species are considered in some category of risk [34]. Furthermore, as in other Mesoamerican bird taxa [44–48], the SMS holds endemic and well-differentiated subspecies, suggesting the importance of isolation for the evolution of intraspecific variation [17, 49].

Isolated montane forest patches are generally thought to act as promoters for divergence between populations of several species, which have served as models for the understanding of speciation processes in island-like settings [50–54]. These fragmented habitats influence the population dynamics and demography, given that resident populations typically show reduced effective population sizes [18, 50, 55–60], they are prone to a reduced adaptive potential and increased extinction risk due to the stochastic effects of genetic drift [61–67]. Therefore, the study of evolutionary history of co-distributed species in these habitats is highly important for conservation purposes.

Herein, we compared phylogeographical structure patterns among four co-distributed and not closely-related Neotropical bird taxa with well-differentiated populations restricted to the SMS. We tested the following biogeographic hypotheses in an ABC framework: (a) a southern origin (southern Central America) followed by a northward expansion; (b) a Mexican origin and range expansion towards southern Central America; and (c) a northern Central American origin with north and southward dispersal. We also aimed to determine whether phylogeographic structure and divergence times in each taxon are congruent among taxa, therefore revealing patterns of simultaneous diversification. Specifically, we used mitochondrial datasets in a comparative phylogeography framework to test: (1) The presence of geographically-structured genetic variation; (2) the existence of spatially-congruent genetic variation across analyzed species; (3) if biogeographic history and similar diversification scenarios support vicariance, dispersal or population admixture. In addition, we also examined temporal patterns of diversification through hABC simulations, testing for either synchronous or multiple pulses for diversification in bird assemblages across the SMS region.

## Results

### Phylogenetic and population structure analyses

**Fig. 2:**
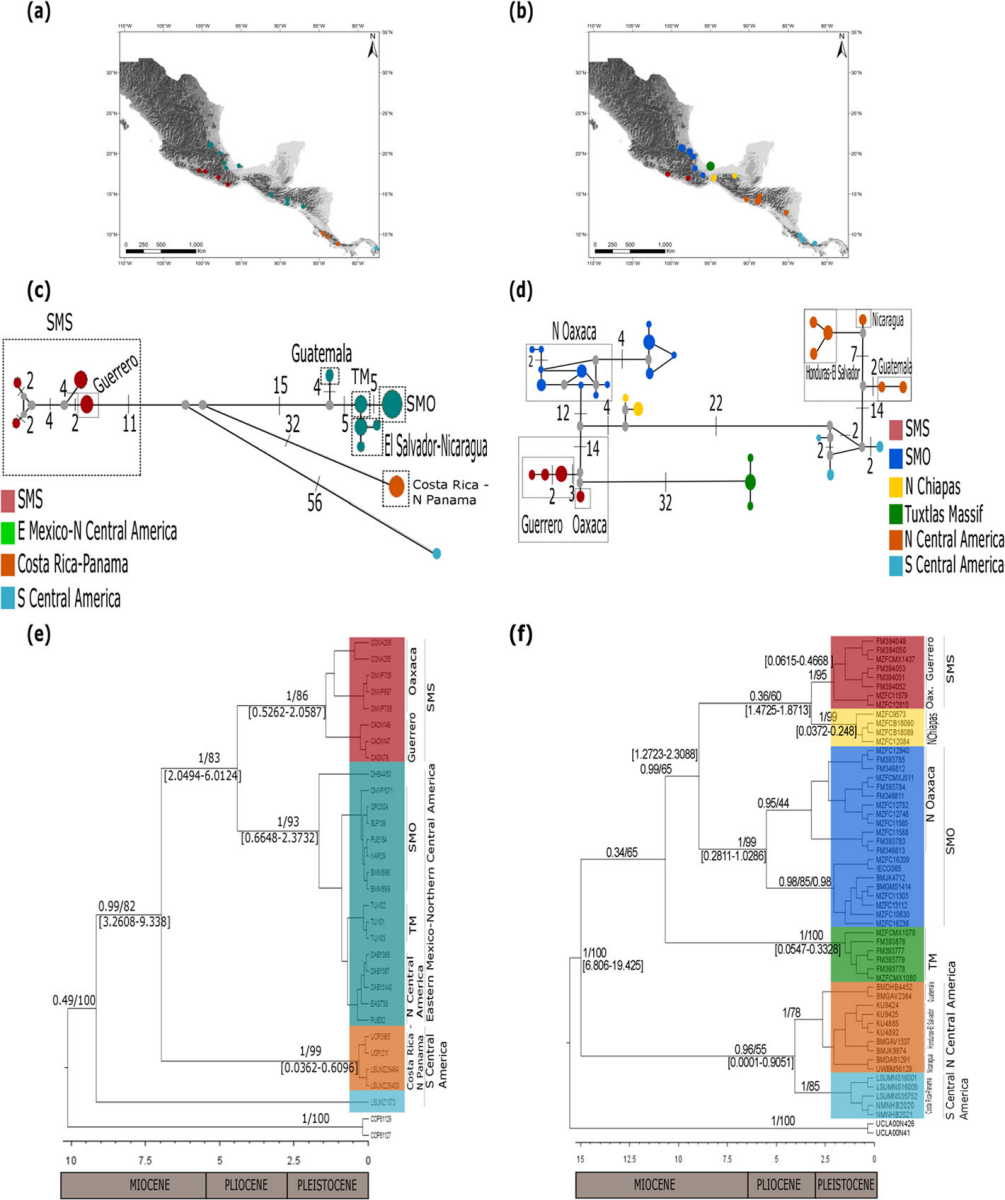
Geographical distribution of sampled haplotypes for (**a**) *Aulacorhynchus* and (**b**) *Chlorospingus*. **c** and **d** depict median-joining network of concatenated mtDNA loci for *Aulacorhynchus* and *Chlorospingus*, numbers on lines depict mutational steps between haplotypes, gray dots represent median vectors inferred for the data. **e** and **f** depict maximum clade credibility trees using BEAST with branch support (BI/bootstrap). Values between brackets indicate the 95% highest posterior densities (HDP) of the estimated times of divergence events (in Myr). Nodes that have no represented time frame of diversification are those whose lower and upper bounds of the HDP interval had posterior probabilities inferior to 0.5

**Fig. 3:**
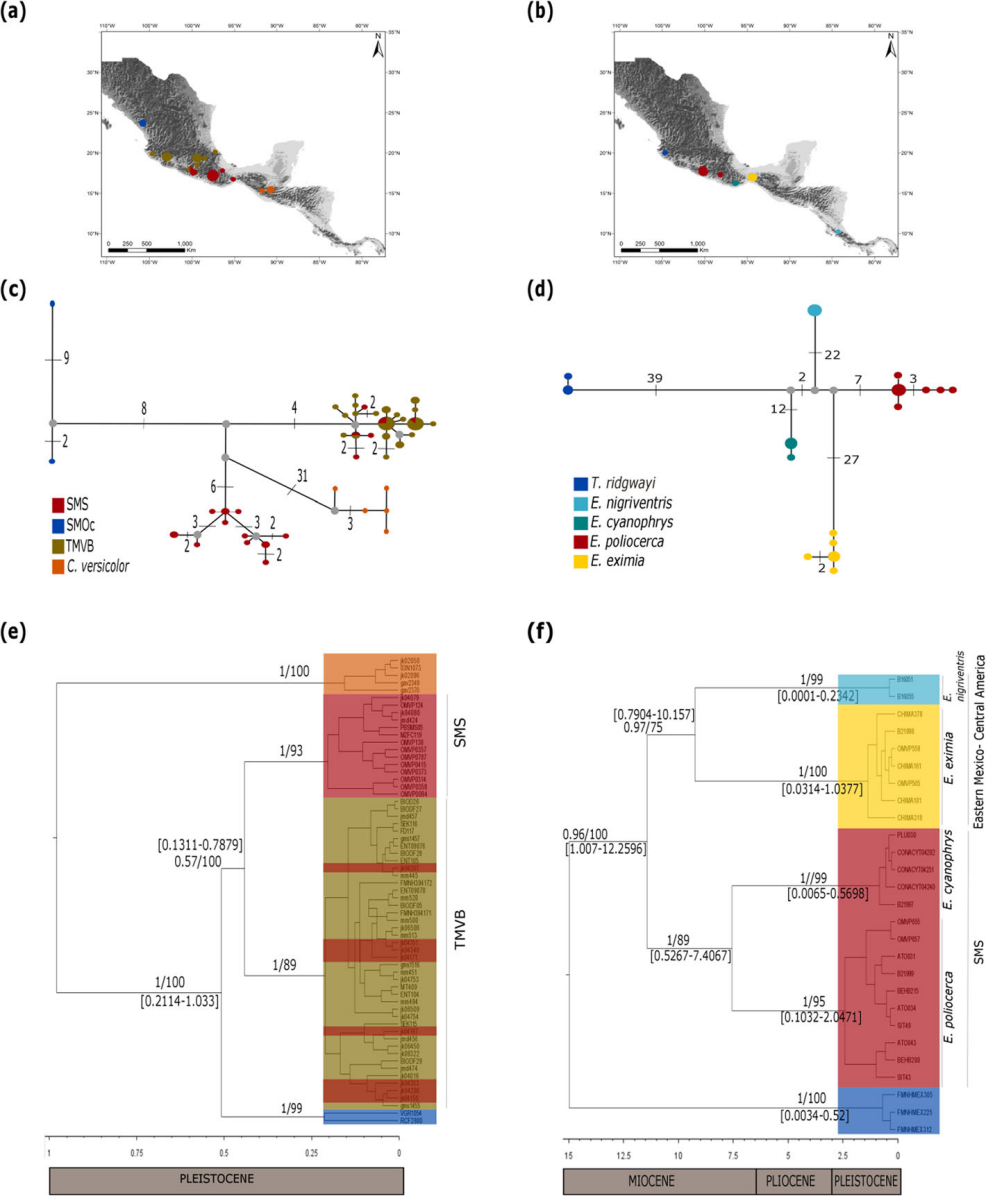
Geographical distribution of sampled haplotypes for (**a**) *Cardellina* and (**b**) *Eupherusa* species. **c** and **d** depict median-joining network of concatenated mtDNA loci for *Cardellina* and *Eupherusa* respectively, numbers on lines depict mutational steps between haplotypes, gray dots represent median vectors inferred for the data. **e** and **f** depict maximum clade credibility trees using BEAST with branch support (BI/bootstrap). Values between brackets indicate the 95% highest posterior densities (HDP) of the estimated times of divergence events (in Myr). Nodes that have no represented time frame of diversification are those whose lower and upper bounds of the HDP interval had posterior probabilities inferior to 0.5

All of our phylogenetic and genetic assignment analyses supported previous findings on the genetic structure for all of the taxa (Fig. 2 and Fig. 3). Genetic structure is clearly delimited by landscape breaks corresponding to the main montane regions in Mexico and Central America. Nevertheless, we found that the detected genetic structure in these taxa is not coincident with the accepted taxonomy at the intraspecific level.

We found four distinctive lineages in *Aulacorhynchus*. Two of these, SMS (restricted to the SMS highlands) and EMx-NCA (widespread in Mesoamerica, including SMO, TM, Chiapas, and northern Central America) are differentiated by 27 mutational steps (Fig. 2c). The other two are distributed in southern Central America from Costa Rica to Panama. Phylogenetic analyses support this structure with ML bootstrap values and BI posterior probabilities above 80 and 0.9 respectively (Fig. 2e). We further observed two subclades in SMS samples, each representing Oaxaca and Guerrero populations.

For *Chlorospingus*, we observed six distinct mtDNA lineages, four of which included Mexican samples (Fig. 2d). Individuals representing the SMS (Sierra de Miahuatlán, Oaxaca and Guerrero) were recovered in a separated group from samples from northern Oaxaca, which clustered with samples from the eastern Sierra Madre Oriental highlands (SMO); both groups are separated by 26 mutational steps. A third group corresponded to the Chimalapas (Oax.) and northern Chiapas region, while the fourth group corresponded exclusively to the TM, separated from the SMS by 32 mutations. This genetic structure is supported by BI, as well as by genetic assignment analyses with overall high posterior probabilities. Some nodes however, showed low bootstrap and posterior probability values (Fig. 2f). As observed in *Aulacorhynchus*, samples from the states of Oaxaca and Guerrero were recovered as distinct subclades within the SMS.

In *Cardellina*, we found three lineages representing *C. rubra* separated by 11 to 24 mutational steps (Fig. 3c). *Cardellina versicolor*, the sister species, is differentiated by more than 30 mutational steps from the *C. rubra* group. Within *C. rubra*, all recovered lineages coincide with previous findings, with one group found in the SMOc, another in the TMVB, and a third one in the SMS. We found 8 samples from a locality in central Guerrero (Carrizal de Bravo) from the SMS to be grouped in the TMVB clade, which also includes samples from localities of Jalisco and Michoacán. This paraphyletic relationship was recovered in all analyses, receiving strong support from both bootstrap (> 80) and Bayesian posterior probabilities (1; Fig. 3e). Nevertheless, genetic assignment analysis yielded a distinct result for these Guerrero samples, particularly for three of them (jk04–349, jk04–351, and jk04–353), which clustered with the SMS group; the remaining five clustered with the TMVB group.

Finally, in *Eupherusa-Thalurania*, we found five lineages corresponding to the currently recognized species. *T. ridgwayi* group is separated by 39 mutational steps from all of the *Eupherusa* samples; species from the SMS *E. cyanoprhys* and *E. poliocerca* haplotypes are separated by 22 mutational steps; and finally, species *E. nigriventris* and *E. eximia* are separated by 50 mutations (Fig. 3d). Topologies from BI, ML (Fig. 3f) recovered high posterior probabilities and bootstrap support values (> 0.8, > 75 respectively). All analyses recovered a monophyletic *Eupherusa* genus, where the SMS is represented by *E. poliocerca* and its sister species *E. cyanophrys*, and a clade from eastern Mexico to Central America, composed of *E. eximia* sister to *E. nigriventris*. Finally, for each of these species, we did not find evidence supporting further intraspecific genetic structure, even when their distribution, as in *E. poliocerca* (Oaxaca-Guerrero) and *E. eximia* (*E. e. nelsoni*, *E. e. eximia*, and *E. e. egregia*), has been described to be disjunct [28, 68, 69].

Hierarchical AMOVAs (Table 1) for the analyzed species showed that variation was explained by differences among groups. In this case, the fixation indices among groups (*F*_CT_) were not significant, whereas fixation indices among populations within groups (*F*_SC_) were. Genetic diversity varied among datasets. Overall, we obtained high haplotype diversity (Hd > 0.5) and low nucleotide diversity (Table 2). The only low value of Hd was observed in *E. cyanophrys*. Fu’s F_S_ and Tajima’s D values were not significant, excepting TMVB and SMS in *Cardellina*; however, we observed a tendency towards negative values (Table 2).

**Table 1:** Hierarchical AMOVAs for each matrix analyzed showing as sources of variation *F*_CT_, *F*_SC_ and *F*_ST_ respectively

Dataset	Groupings	Source of variation	Sum of squares	Variance components	Percentage of variation	Statistics	Significance (p)
*Aulacorhynchus*	(NP) (CR-NP) (EMx-NCA) (SMS)	Among groups	368.978	19.644	84.62	*F*_*CT*_ = 0.846	0.091
		Among populations within groups	8.928	1.382	5.96	*F*_*SC*_ = 0.387	< 0.00001
		Within populations	52.51	2.187	9.42	*F*_*ST*_ = 0.905	< 0.00001
*Chlorospingus*	(TM) (N Chiapas) (SMO) (SMS) (NCA) (SCA)	Among groups	702.506	15.108	79.51	*F*_*CT*_ = 0.795	0.053
		Among populations within groups	7.792	1.949	10.26	*F*_*SC*_ = 0.5	0.041
		Within populations	89.4	1.943	10.23	*F*_*ST*_ = 0.897	< 0.00001
*Cardellina*	(TMVB) (SMS) (SMOc) (*C. versicolor*)	Among groups	195.29	12.94493	71.09	*F*_*CT*_ = 0.71094	0.1564
		Among populations within groups	69.803	2.5099	13.78	*F*_*SC*_ = 0.47687	< 0.00001
		Within populations	162.447	2.753	15.12	*F*_*ST*_ = 0.84879	< 0.00001
*Eupherusa*	(*E. nigriventris)* (*E. cyanophrys*) (*E. poliocerca*) (*E. eximia*) (*T. ridgwayi*)	Among groups	392.306	18.846	93.16	*F*_*CT*_ = 0.9314	0.077
		Among populations within groups	4	0.654	3.23	*F*_*SC*_ = 0.472	0.1886
		Within populations	15.324	0.729	3.61	*F*_*ST*_ = 0.963	< 0.00001

**Table 2:** Summary statistics of each analyzed species populations, based on K groups observed in phylogenetic and Geneland analyses, using concatenated mtDNA. *N* = number of sequences, Hd = haplotype diversity, π = nucleotide diversity. Fu’s F_S_ and Tajima’s D values with an asterisk (*) represent significant values for their respective confidence intervals (< 0.02 and <  0.05). Columns with “n. a.” represent null genetic variation owing to the presence of only one haplotype. Region abbreviations are as follows: TMVB = Trans Mexican Volcanic Belt, SMS = Sierra Madre del Sur, SMOc = Sierra Madre Occidental, SMO = Sierra Madre Oriental, NCA = northern Central America, SCA = southern Central America, EMx-NCA = eastern Mexico-northern Central America, CR-NP = Costa Rica-northern Panama, SP = southern Panama

Diversity Indexes	Species																		
	*Cardellina*				*Chlorospingus*						*Aulacorhynchus*				*Eupherusa*				
Groups	*Cversicolor*	TMVB	SMS	SMOc	SMO	SMS	Tuxtlas Massif	N Chiapas	NCA	SCA	SMS	EMx-NCA	CR-NP	SP	*T. ridgwayi*	*eximia*	*poliocerca*	*cyanophrys*	*nigriventri*
*N*	5	42	14	2	20	8	6	4	10	5	8	11	4	1	3	7	10	5	2
Hd (S. D.)	1 (0.1265)	0.982 (0.046)	0.967 (0.036)	1 (0.5)	0.931 (0.034)	0.821 (0.1007)	0.6 (0.215)	0.5 (0.265)	0.844 (0.1029)	0.8 (0.164)	0.785 (0.112)	0.5636 (0.134)	n. a.	n. a.	0.66 (0.314)	0.857 (0.137)	0.77 (0.137)	0.4 (0.237)	n. a.
π (S. D.)	0.00126 (0.00092)	0.00264 (0.0015)	0.0021 (0.0012)	0.0046 (0.0048)	0.0083 (0.0047)	0.00518 (0.0033)	0.00104 (0.00105)	0.00234 (0.002)	0.0084 (0.005)	0.0095 (0.0062)	0.0055 (0.0033)	0.0045 (0.0026)	n. a.	n. a.	0.0006 (0.0007)	0.0015 (0.00119)	0.0024 (0.0016)	0.0004 (0.0005)	n. a.
Tajima’s D	−0.74682	−1.732*	−0.686	n. a.	0.5083	1.0958	−1.131	−0.75445	−0.20935	0.9544	0.734	−0.33	n. a.	n. a.	n. a.	−1.023	0.138	−0.816	n. a.
Fu’s F_S_	−2.2375*	−17.67*	−3.874*	2.397	−2.84	1.226	−0.858	1.716	0.57	3.1833	2.75	5.29	n. a.	n. a.	0.2	−2.019	−1.206	0.09	n. a.

Overall, pairwise *F*_ST_ values showed significant high genetic differentiation between analyzed groups. However, probably due to small sample sizes, some comparisons showed no significant values, as in the SP cluster of *Aulacorhynchus* (Table 3), as well as in comparisons between *E. nigriventris* vs *T. ridgwayi*, and *E. nigriventris* vs *E. cyanophrys* clusters (Table 4). The lowest *F*_ST_ value obtained occurred between *Cardellina* clusters from the TMVB vs SMS (Table 4).

**Table 3:** Pairwise *F*_ST_ fixation index used to measure genetic structuring in *Aulacorhynchus* and *Chlorospingus*. Abbreviations correspond to defined geographical groups: EMx-NCA (eastern Mexico-northern Central America), CR-NP (Costa Rica-northern Panama), SP (southern Panama), SMS (Sierra Madre del Sur), SMO (Sierra Madre Oriental), NCA (northern Central America), SCA (southern Central America). An asterisk (*) represents significant values (< 0.05)

	EMx-NCA	CR-NP	SP	SMS	SMO	Tuxtlas Massif	*N* Chiapas	NCA	SCA
*Aulacorhynchus*									
EMx-NCA	–	0.923*	0.922	0.849*					
CR-NP		–	1	0.907*					
SP			–	0.904					
SMS				–					
*Chlorospingus*									
SMS				–					
SMO				0.863*	–				
Tuxtlas Massif				0.941*	0.912*	–			
N Chiapas				0.905*	0.839*	0.976*	–		
NCA				0.894*	0.877*	0.911*	0.882*	–	
SCA				0.885*	0.886*	0.928*	0.893*	0.784*	–

**Table 4:** Pairwise F_ST_ fixation index used to measure genetic structuring in *Cardellina* and *Eupherusa*. Abbreviations correspond to defined geographical groups: SMOc (Sierra Madre Occidental), TMVB (Trans Mexican Volcanic Belt), SMS (Sierra Madre del Sur). An asterisk (*) represents significant values (< 0.05)

	*C. versicolor*	SMOc	TMVB	SMS	*T. ridgwayi*	*E. nigriventris*	*E. eximia*	*E. poliocerca*	*E. cyanoprhys*
*Cardellina*									
*C. versicolor*	–								
SMOc	0.9003*	–							
TMVB	0.93116*	0.82625*	–						
SMS	0.80247*	0.5419*	0.47295*	–					
*Eupherusa*									
*T. ridgwayi*					–				
*E. nigriventris*					0.991	–			
*E. eximia*					0.975*	0.959*	–		
*E. poliocerca*					0.952*	0.926*	0.941*	–	
*E. cyanoprhys*					0.99*	0.991	0.972*	0.917*	–

### Divergence time estimation and coalescent-based estimation of population history

**Fig. 4:**
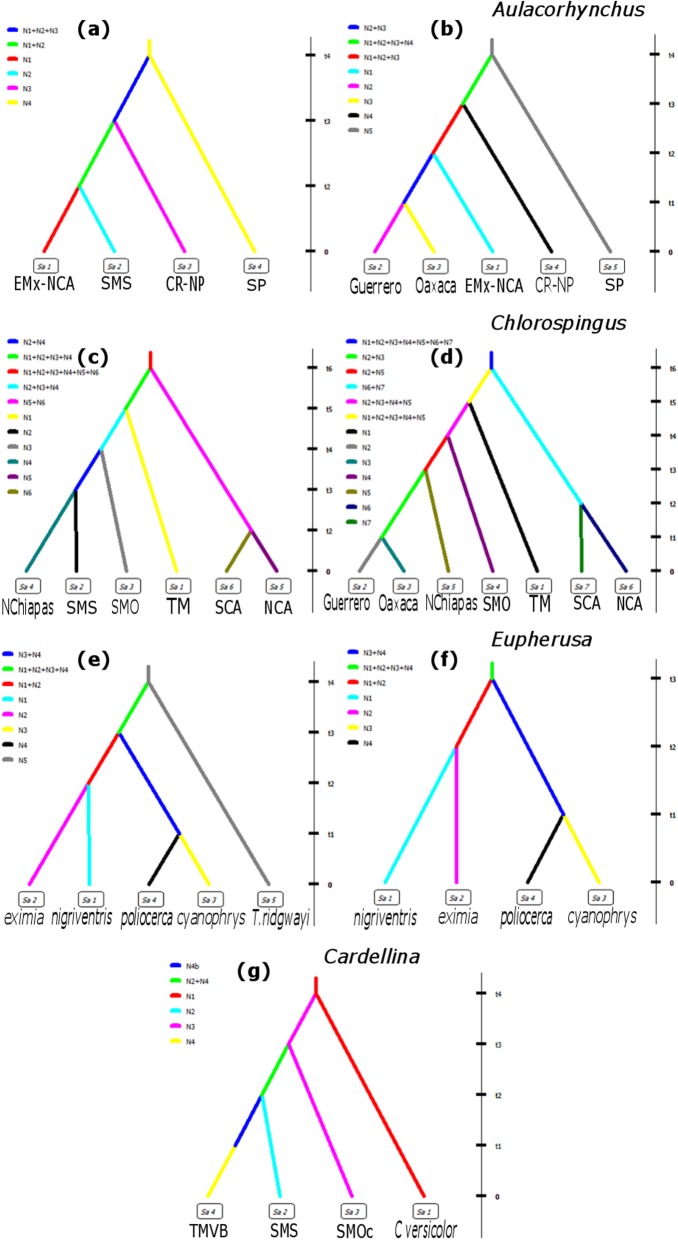
Best-supported biogeographic scenarios of diversification in analyzed species. **a** scenario 1 of diversification for *Aulacorhynchus*. **b** Scenario 2 of diversification for *Aulacorhynchus* considering as distinct populations lineages from Oaxaca and Guerrero. **c** Scenario 1 of diversification of *Chlorospingus* species. **d** best-supported scenario in *Chlorospingus* considering lineages of Guerrero and Oaxaca as distinct populations. **e** Scenario 2 of diversification in *Eupherusa* species including *T. ridgwayi*. **f** Scenario 1 of diversification in *Eupherusa* excluding *T. rigdwayi*. **g** Scenario 3 of diversification in *Cardellina* species

Even when divergence dates varied, analyses showed that divergence between populations occurred during the Pleistocene, mainly within the last 2 Myr. The best-supported scenario for four population groups in *Aulacorhynchus* was scenario 1 (Additional file 5: Figure S5), with a posterior probability (PP) of 0.57 and a 95% confidence interval (CI) of 0.5584–0.5816 (Fig. 4a), which suggested that SMS, EMx-NCA, and CR-NP populations arose from an ancestral SP population, following a northwards divergence pattern. Confidence in scenario choice tested through type I and II errors, suggested the selection of a highly accurate scenario (Additional file 3). Posterior parameter estimates (Table 5) indicated that initial divergence (*t4*) from southern Panama northwards across the lowlands and narrowest region in Central America (Panama Channel Fault Zone) started during the Pliocene at 4.3 Myr (95% CI: 4–4.8 Myr), followed by successive northward expansion and further vicariance of populations, and the final divergence between eastern (EMx-NCA) and southwestern (SMS) Mexican populations (*t2*) about 1.3 Myr (95% CI: 420 kyr – 2.5 Myr) during the Pleistocene. When the lineages of Guerrero and Oaxaca were considered as distinct populations (Additional file 5: Figure S6), the best scenario was scenario 2 (PP of 0.677, 95% CI: 0.668–0.687; Fig. 4b). Type I and II errors indicated moderate confidence in the selected scenario (Additional file 3). Under this scenario, diversification patterns were similar to the latter, but the most recent divergence event occurred between Oaxaca and Guerrero populations (Additional file 4 and Additional file 5). Mean posterior parameter estimates (Table 5) indicated that the first divergence (*t4*) event is dated around 3.3 Myr (95% CI: 2.5–6.1 Myr), while the most recent divergence time (*t1*) occurred about 204 kyr (95% CI: 12.8–730 kyr).

**Table 5:** Mean time posterior parameter estimates for the best supported scenarios of analyzed species. Estimates are based on the 1% of simulated datasets closest to the observed values. Time (*t*) is set in years

Dataset	Groupings	*t1*	*t2*	*t3*	*t4*	*t5*	*t6*
*Aulacorhynchus*	Four population groups		1,328,000	2,860,000	4,340,000		
	Five population groups	204,000	1,884,000	2,300,000	3,300,000		
*Chlorospingus*	Six population groups		1,308,000	2,060,000	2,840,000	3,900,000	5,860,000
	Seven population groups	656,000	1,014,000	1,218,000	1,598,000	1,946,000	2,040,000
*Cardellina*		40,200	260,000	426,000	1,304,000		
*Eupherusa*	Including *T. ridgwayi*	716,000	1,346,000	1,804,000	2,780,000		
	Excluding *T. ridgwayi*	688,000	1,290,000	1,724,000			

The best scenario for *Chlorospingus* when six groups are considered (Additional file 5: Figure S7), was scenario 1 (PP = 0.519, CI: 0.4965–0.5427; Fig. 4c). Confidence in scenario choice was high given the values for type I and II errors (Additional file 3). This scenario suggested an initial divergence (*t6*) of Mexican and Central American lineages during the Pliocene at 5.8 Myr (95% CI: 2.5–9.5 Myr (Table 5), diversification of Mexican lineages proceeded westward, with the latter divergence event (*t2*) between NCA and SCA at 1.3 Myr (95% CI: 138 kyr – 2.7 Myr; Additional file 4 and Additional file 5). When lineages from Oaxaca and Guerrero were considered as distinct populations, scenario 1 (Additional file 5: Figure S8) received the better support (PP = 0.6, 95% CI: 0.5909–0.625; Fig. 4d), with high confidence (Additional file 3). Scenario 1 suggested diversification events similar to the latter, mean parameter estimates (Table 5) suggested an initial divergence time (*t*6) at 2 Myr (95% CI: 1.1–5.3 Myr) between Mexican and Central American lineages, a divergence event (*t*2) estimated to occur after the Nicaragua Depression formation between Central American populations at 1 Myr (95% CI: 288 kyr – 2.1 Myr), and a final divergence event (*t1*) in the SMS lineage originating Guerrero and Oaxaca populations during a interglacial period in the pre-Illinoian stage at 656 kyr (95% CI: 50 kyr – 1.6 Myr; Additional file 4 and Additional file 5).

Best-supported scenario in *Cardellina* was scenario 3 (PP = 0.4414, CI: 0.43–0.45; Fig. 4g), with high confidence (Additional file 3). Scenario 3 (Additional file 5: Figure S9) predicts an initial northward dispersal event from northern Central America (*C. versicolor*) population through the Isthmus of Tehuantepec at *t4* (~ 1.3 Myr); followed by vicariance events driven by the effect of Mexican highlands at *t3* (~ 426 kyr) between SMOc and an hypothetical ancestral population that at *t2* (~ 260 kyr) is fragmented into the TMVB and SMS populations (Table 5). This scenario also supports a recent change in the TMVB effective population size during the Wisconsin Glacial stage at 40 kyr (95% CI: 3500 yr – 122 kyr; Additional file 4 and Additional file 5).

The best scenario for *Eupherusa* including *T. ridgwayi* (Additional file 5: Figure S10) was scenario 2 (PP = 0.712, 95% CI: 0.7–0.72; Fig. 3e). Type I and II errors indicate high confidence in scenario choice (Additional file 3). Scenario 2 suggested divergence of *T. ridgwayi* and a hypothetical ancestral population during the late Pliocene at (*t4*) 2.7 Myr (95% CI: 2.1–3.4 Myr), in time (*t3*) and before major climatic oscillations in the mid-Pleistocene, the ancestral *Eupherusa* lineage splits into two hypothetical ancestral lineages, one that further splits at *t2* (~ 1.3 Myr) giving rise to the populations in eastern Mexico and Central America (*E. eximia*–*E.nigriventris*), and the second which splits into populations in the SMS (*E. poliocerca-E. cyanophrys*) at 716 kyr (95% CI: 123 kyr – 1.5 Myr; Table 5). When *T. ridgwayi* is excluded (Additional file 5: Figure S11), the best-supported was scenario 1 (PP = 0.868, 95% CI: 0.859–0.877; Fig. 4f), which is similar to the previous scenario. Confidence in scenario choice was high given low values of type I and II errors (Additional file 3). Mean parameter estimates of scenario 1 (Table 5) indicated an initial divergence at *t3* (~ 1.7 Myr) of an ancestral *Eupherusa* lineage into two ancestral populations. One diverged into eastern Mexican and Central American populations at *t2* (~ 1.2 Myr), whereas the other splits into southwestern Mexican populations at 688 kyr (95% CI: 116 kyr – 1.5 Myr; Additional file 4 and Additional file 5).

### Test of simultaneous divergence

**Fig. 5:**
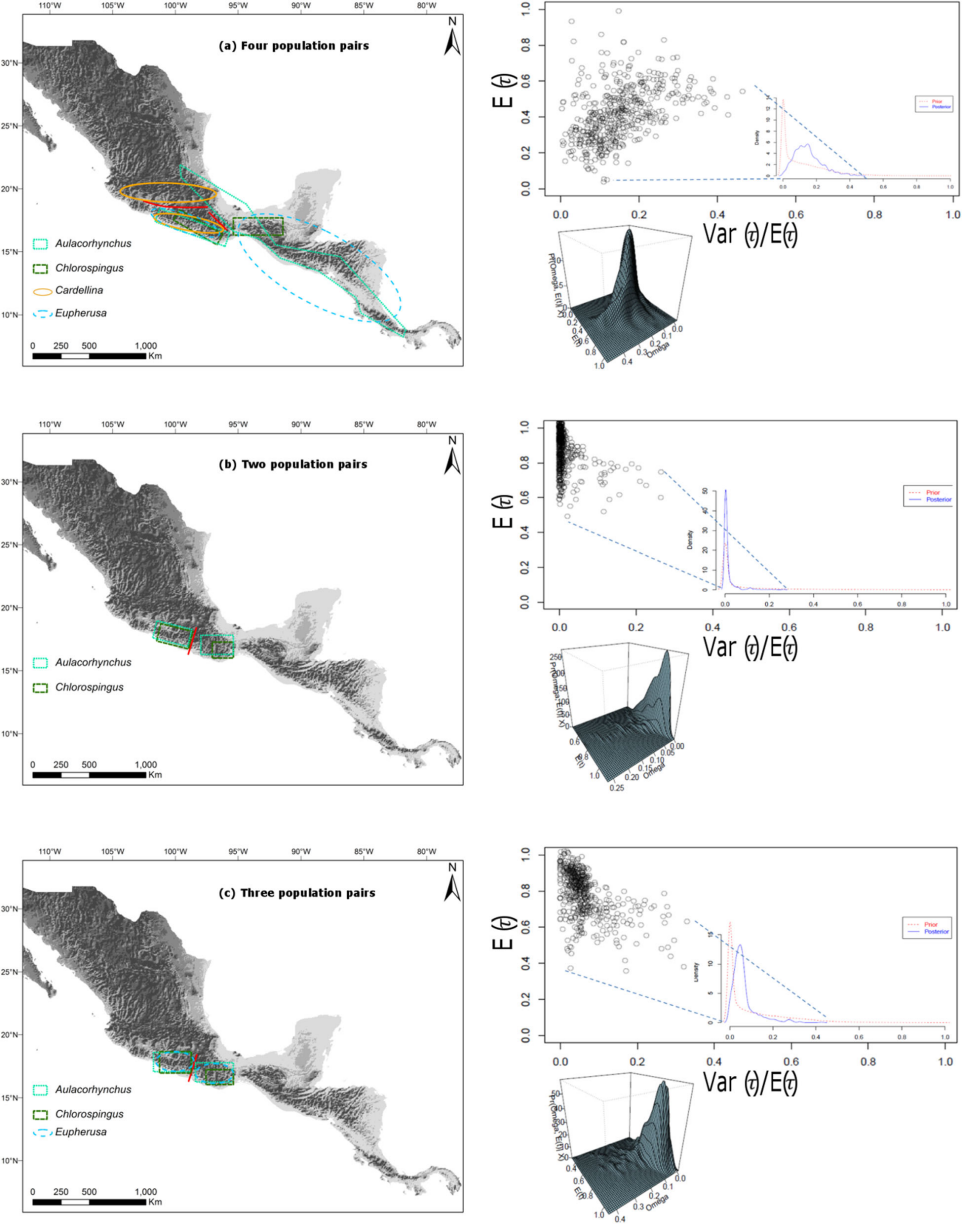
Joint posterior probability for *E*(τ) (average divergence time) and Ω (variance in divergence times/average divergence time) for (**a**) Four population pairs spanning across southwestern Mexico and eastern-central Mexico through Central America. **b** Two population pairs within SMS. **c** Three population pairs within SMS

The hABC analyses including four population pairs spanning along two identified putative barriers (Balsas Depression and Oaxaca’s Valleys)*,* showed a relatively strong posterior probability support for four independent divergence events (PP_Ψ = 4_ = 0.417) and the highest mode model-averaged posterior estimate of the hyperparameter Ω = 0.1415 (95% HDP interval = 0.0035–0.2998), in comparison with posterior probability of two (PP_Ψ = 2_ = 0.282) or three (PP_Ψ = 3_ = 0.275) divergence events (Fig. 5a).

Subsequently, we tested subsets of the data set for simultaneous divergence. First we tested two taxon pairs within SMS (Guerrero and Oaxaca populations separated by the Río Verde drainage) of *Aulacorhynchus* and *Chlorospingus*, obtaining high support for a single divergence event (PP_Ψ = 1_ = 0.6749), along with a mode value of Ω consistent with synchronous divergence (Ω = 0.000475) and an Ω = 0, which is included within the 95% HDP intervals (Fig. 5b), whereas when including the *Eupherusa* taxon pair (*cyanophrys-poliocerca*), we found low support for a single divergence event, suggesting two divergence events across these taxa (PP_Ψ = 2_ = 0.5436) and a Ω mode of 0.05 consistent with asynchronous divergence (Fig. 5).

**Fig. 6:**
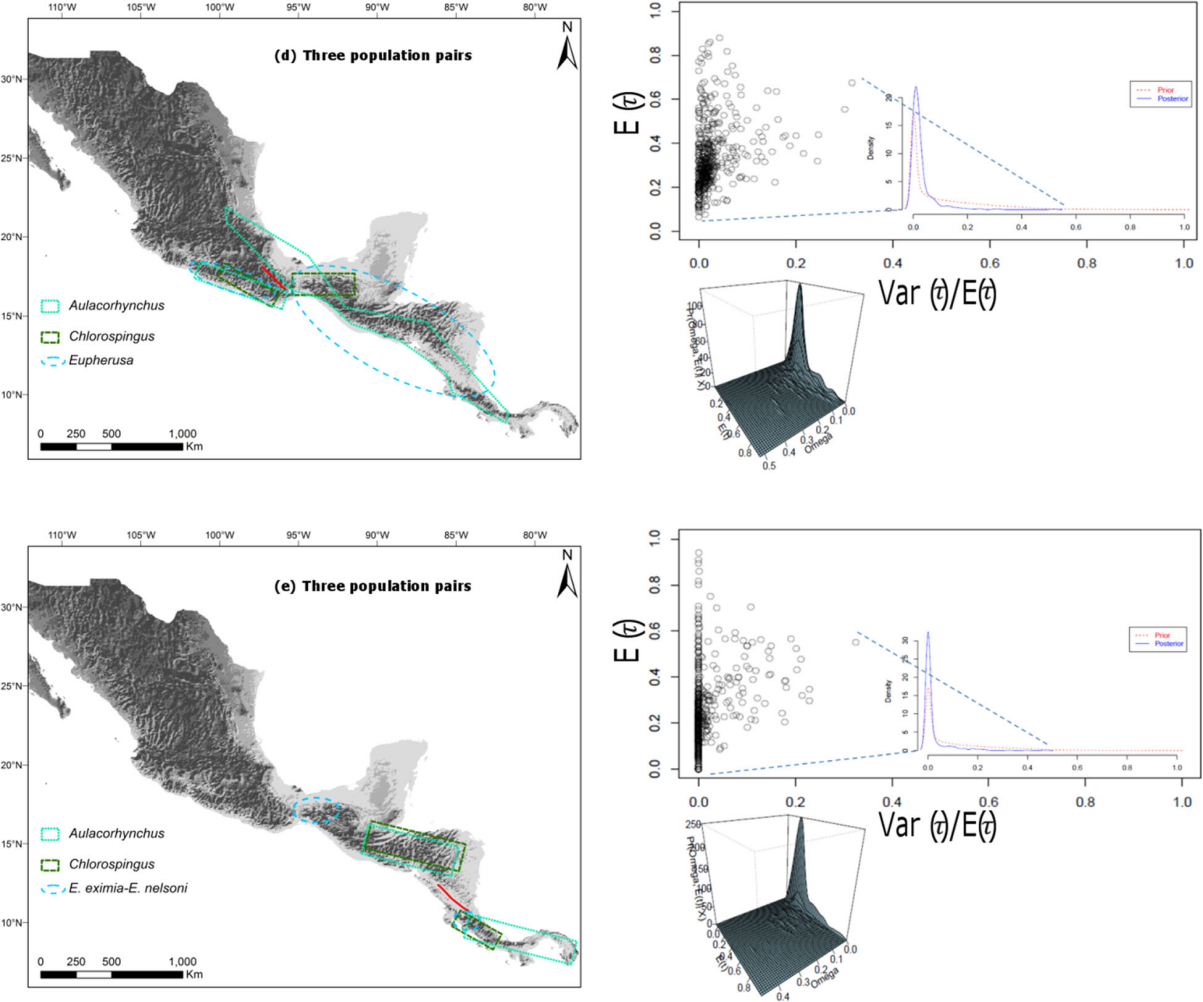
Joint posterior probability for *E*(τ) (average divergence time) and Ω (variance in divergence times/average divergence time) for (**d**) Three population pairs spanning across southwestern Mexico and eastern Mexico through Central America. **e** Three population pairs in Central America

When evaluating southwestern SMS against eastern populations spanning Oaxaca’s Central Valley barrier, there was a relatively strong support for a single divergence event between these populations (PP_Ψ = 1_ = 0.4536) and Ω = 0.0022, also indicating support for synchronous diversification between these taxa occurring at ~ 1.06 Myr (Fig. 6d). Finally, the evaluation of population pairs distributed along Central America revealed a very high posterior probability of one divergence event (PP_Ψ = 1_ = 0.7228), and a Ω hyperparameter value = 0 (95% HDP interval = 0.0–0.1167) across the Nicaraguan Depression lowlands between Costa Rica and Nicaragua occurring ~ 0.924 Myr (Fig. 6e).

## Discussion

The aim of comparative phylogeography is to detect concordant patterns among co-distributed species, based on the idea that they may share common histories [1, 14, 16]. Thus, finding similar genetic patterns among different species may suggest similar historical processes influencing major biological components in a given region. Nevertheless, as shown in the results presented herein, complete concordance is commonly non-existent, which is probably a result of intrinsic characteristics of species (e.g., feeding habits) triggering different responses to particular events, therefore emphasizing the role of idiosyncratic events in the recent evolutionary history of genetic loci and bird taxa in the region [14, 58].

Furthermore, altitudinal gradients may shape broad biogeographical patterns and potentially population genetic variation but its influence is complex and variable among species [70–73]. Models show that cold and humid conditions were extended along the Mexican highlands during the Pleistocene; nevertheless the dynamics under which each taxon responded to the climatic cycles depends on several factors (e. g., time of establishment in an area, environmental preferences, dispersal capabilities [74]). Our study also indicates that at least two unrelated bird genera (*Aulacorhynchus* & *Chlorospingus*) with distinct natural histories but which inhabit roughly the same regions and wide elevational range along the humid montane forests of Mesoamerica, present highly similar phylogeographic structure, including a significant split between the highlands in Guerrero and Oaxaca within the SMS, which may be expected due to discontinuous distribution ranges, as well as to their presumably reduced dispersal abilities. The pattern observed in *Cardellina* and *Eupherusa* do not fully agree with the expectation that the degree of isolation between montane ranges is responsible for shaping the genetic differentiation between populations, as has been reported in previous studies of montane birds (see [45, 75]). The analyses in these two taxa evidenced exclusive haplotypes from Oaxaca, but genetic differentiation across the Río Verde drainage, as seen in *Aulacorhynchus* and *Chlorospingus* does not occur. Regarding *Cardellina*, despite the fact that the TMVB and the SMS montane ranges are separated by a significant geographic barrier such as the Balsas Depression (see [7]), incomplete lineage sorting between both regions is reported. These differences may also be due to the fact that both taxa inhabit different elevational ranges along the region: *Eupherusa* ranging to the lower limits of the cloud-forest and *Cardellina* ranging in the upper reaches of the humid montane forest, suggesting that isolation and connectivity cycles in the vegetation types might have a differential impact on the genetic pool of birds depending on the elevational range, which might explain the observed patterns [74].

### Phylogenetic and population structure analyses

Our results, as several others from the Neotropics (see [11, 13, 18, 76, 77]), found shared phylogeographic breaks at which genetic differentiation occurs among widespread Mesoamerican highland bird species. We observed major breaks, as seen in *Aulacorhynchus* and *Chlorospingus*, between Chiapas and Mexican-Central American lineages. This has been also supported by other bird species [75], plants [18], rodents [20, 35], insects [51] and a snake [78]. Moreover, phylogeography shows isolation between populations inhabiting the mountains of southwestern Mexico (SMS) and northern Oaxaca (see *Chlorospingus*) evidencing that populations are clearly sorted within Oaxaca across its Central Valley, and more importantly morphological variation of these populations has also been documented as in *E. eximia nelsoni* [79]. Isolation between Central American highland populations (NCA-SCA) has been likely promoted by the Nicaraguan Depression (see [13, 77, 80]). The most relevant break in our study, occurred within the SMS region, between the states of Oaxaca and Guerrero highlands, which are separated by the Río Verde drainage, this break is supported by studies in plants [33], lizards [81, 82], frogs [31] and even in lowland species, such as iguanas [83].

The patterns observed in the haplotype networks mostly reveal a consistent match between haplogroups and subspecies, as well as species boundaries (see *Eupherusa*). The overall structures in the haplotype networks resemble an expected pattern for ancient divergence among the sky-islands where reciprocally monophyletic groups occur on each sky-island with no mixing (see *Aulacorhynchus* and *Chlorospingus*), and is consistent with estimated divergence and the gradual cooling occurring in the Gelasian stage of late Pliocene-early Pleistocene [84]; whereas in *Eupherusa* it is difficult to discern specific events for each divergence estimate due to wide confidence intervals. *Cardellina* is an exception which resembles a star-shaped network of a post glacial sky-island divergence [53] which is consistent with an interglacial period during the Pre-Illinoian stage [84, 85] and the estimated divergence in BEAST and DIYABC analyses.

AMOVA results highlighted the existence of genetic structure for all studied species. Most molecular variation was found among groups; nevertheless, the fixation indices (*F*_SC_) among populations within groups were significant, as in *Aulacorhynchus*, *Chlorospingus* and *Cardellina*. This pattern suggests the existence of further structured populations in at least one of the defined clusters, as supported by haplotype networks (Fig. 2b and Fig. 3d).

We obtained overall high values of haplotype diversity (Hd) and low nucleotide diversity (π) for the majority of analyzed groups within each taxon. These patterns have been attributed to population growth following demographic bottlenecks enhancing retention of novel mutations [86, 87], which is congruent in sky-island populations supporting lower nucleotide variation given reduced gene flow to other populations [52, 88, 89]. Population expansions may have been promoted by climatic oscillations during the Pleistocene; nevertheless, we found significant support for population expansion only in *Cardellina* populations from the TMVB, and SMS. For the rest of the taxa, Tajima’s D and Fu’s F_S_ values suggest that the observed nucleotide polymorphism is selectively neutral even when they tend towards negative figures, thus favoring demographic stability during the Pleistocene. Pairwise comparisons of *F*_ST_ values for each taxon are high and mostly significant, which is expected given their disjunct geographic distribution. Overall, all populations concerned are allopatric and several lineages possess phenotypic and phylogenetic identity, thus the *F*_ST_ fixation indices support the idea that these populations have followed their own evolutionary trajectory for a long time (Tables 3, 4). Despite the *F*_ST_ pairwise comparison between SMS and TMVB populations was low; according to [90] these populations have great genetic differentiation. Moreover, some populations, despite allopatry, may be geographically close enough to show evidence of intermittent connections in response to environmental changes [91].

### Divergence time estimation and coalescent-based estimation of population history

Our results highlight the importance of Pliocene-Pleistocene events in promoting the intra-specific genetic structure in the analyzed species, as reported in other studies. Divergence between SCA and northern populations is concordant with the formation of the Nicaragua Depression (ca. 4 Myr [92];), furthermore, divergence between NCA and Mexican lineages post-date the time frame of the formation of the volcanic arcs in Central America (Miocene-Pliocene or before [92];), the IT dry plains (ca. 6–3 Myr [38];), orogeny of SMS (ca. 35–20 Myr), and the formation of the TMVB as well as its period of volcanism in central Mexico (ca. 16–7 Myr [39–41, 93, 94];). Our results suggest that these geographic breaks may have acted indirectly as semi-permeable barriers to dispersal, leading to an accumulation of processes involving isolation and expansion [95], as the *Cardellina* analyses revealed.

DIYABC scenarios allowed us to infer evolutionary processes affecting populations. Although estimated divergence dates varied among populations of the analyzed taxa, major splits occurred during the Pleistocene. Scenarios from *Aulacorhynchus*, *Chlorospingus*, and *Cardellina*, along with their respective pairwise *F*_ST_ comparisons, support the hypothesis of sequential northward population range expansions from Central America with ancestral populations being split by vicariant events, such as fracture and changes in altitudinal ranges of montane forests linked to climatic oscillations, therefore promoting geographical isolation. Although our analyses support a southern origin, the pattern observed in *Eupherusa* + *Thalurania ridgwayi* suggest alternative scenarios as also probable. Based on previous evidence supporting *T. ridgwayi* as a member of the *Eupherusa* genus [96, 97], we included samples of *T. ridgwayi* in the diversification scenario comparisons, and we were unable to discern if diversification according to the best-supported scenario occurred from two potential regions that may have served as refugia (one in northwestern Mexico, another in southern Mexico-Central America), or whether SMS (*E. poliocerca-E. cyanoprhys*) and Central American lineages of *Eupherusa* come from a migration event from northwestern Mexico. Further studies should focus on the reconstruction of ancestral areas of distribution to clarify this issue (see [50]).

Overall, dates from the DIYABC analyses suggest more recent diversification times than our BEAST analyses, thus highlighting the effect that distinct assumed mutation rates have an effect of temporal frame estimation [98]; nevertheless, these values overlap their respective 95% HDP confidence intervals. Even in vicariant events, differences in time estimates can also be due to variance in the coalescent process and related to the demography of each species [18, 50], however we acknowledge the inconveniences of single locus based analyses and that distinct calibration for divergence time estimation may yield potential errors, thus future surveys incorporating more data should be able to obtain stronger and more accurate estimates of timing and synchrony of divergence.

### Test of simultaneous divergence

Even when species ranges overlap across their distributional areas, some divergence time estimation frames are not congruent with each other, therefore, asynchrony in diversification within Mesoamerica could be depicted as a result of Pleistocene climatic oscillations which promoted rapid pulses of diversification and speciation rates across multiple lineages [13, 18, 19]. Our analyses including four population pairs detected non-simultaneous divergence events within the Pleistocene, pointing to the existence of several temporal frames where diversification and fixation of genetic differences within the same montane ranges occurred among distinct lineages. Our results also highlight a high probability of simultaneous divergence occurrence between two taxa (*Aulacorhynchus* and *Chlorospingus*) in the SMS region across the Río Verde drainage lowland barrier, with a mean divergence date of 0.9339 Myr, which 95% HDP confidence intervals falls within the estimated divergence dates between the same populations. When *Eupherusa* population pair (*cyanophrys-poliocerca*) was added, estimations supported two diversification events, thus implying that even when the three taxa share their overall distributional ranges in the SMS, they do not fully share a community-wide historical pattern across the landscape.

Even when we do not fully out rule any orogenic effect over the analyzed species, major changes to population structure should be promoted by the onset of climatic oscillations in the Pleistocene, which have been invoked to explain patterns of genetic differentiation in the increasing rates of speciation and diversification in Neotropical biota, given the evidence of a downward altitudinal migration of the forest line which resulted in a montane forest-like dominated landscape in the lowlands with further retreat when temperatures raised [99–101]. Although for the *C. rubra* lineage the observed strong signature of expansion in TMVB and SMS populations due to Pleistocene conditions and forest migrations resulted in the admixture of two isolated lineages; our results reveal that demographic dynamics for other species persisted in isolated regions throughout the Pleistocene allowing the evolution of high endemism, which could be an overall result of high effective population sizes.

Genetic endemism as a result of isolation of intraspecific lineages, as seen within the SMS should be considered in conservation measures given that they preserve historical components and maintain the species capability of adaptation [3, 66].

## Conclusions

Our survey provides a framework for understanding the complex geographic history of Mesoamerica and its role in promoting lineage diversification in birds. From our analyses we recover multiple independently evolving lineages restricted to montane areas across the region, our results also highlight that congruence may be difficult to occur given idiosyncratic life histories of species; nevertheless, co-distributed taxa will share more common history than a random scenario could predict. Montane regions within Mesoamerica are strong drivers of lineage diversification, and as such our results point into major areas of great genetic diversity and endemism, such as isolated highlands of the SMS in the Mexican states of Oaxaca and Guerrero. Given ancient development of major montane systems, climatic oscillations and orogeny derived conditions were key drivers of diversification between these lineages as splits were calculated to occur mainly within the Pleistocene.

## Methods

### Sampling and sequence data

We selected four resident and co-distributed avian taxa from the montane forests of Mesoamerica, all of which represent different orders and families (Apodiformes: Trochilidae; Piciformes: Ramphastidae; and Passeriformes: Parulidae and Passerellidae), as well as life histories. Selected taxa include Toucanets (genus *Aulacorhynchus*), the Red and Red-faced Warbler (genus *Cardellina*), Bush-tanagers (genus *Chlorospingus*), and hummingbirds (genus *Eupherusa-Thalurania*). These taxa include at least one differentiated population restricted to the SMS for which we have previous genetic and morphometric information [5, 28, 68, 69, 96, 97, 102–109]. These studies have also estimated similar Pliocene-Pleistocene divergence times for all of the analyzed taxa.

### Phylogenetic and population structure analyses

For each taxon, we retrieved mitochondrial DNA (mtDNA) sequences deposited in GenBank (https://www.ncbi.nlm.nih.gov/genbank/), and conducted analyses using alignments of concatenated mtDNA loci (Additional file 1). We estimated nucleotide substitution models and partition schemes for each species in PARTITIONFINDER [110], using the Bayesian Information Criterion (BIC) for model selection. Given the10 bp frameshift overlap in the ATPase 8 and 6 genes and the tRNA-Lys gene, we analyzed these as a single genetic region [111, 112]. Resultant partition schemes and model substitution parameters were used for conducting a phylogenetic reconstruction using the Bayesian inference approach (BI) for each species, as implemented in MRBAYES 3.2 [113]. We ran two independent searches using four Markov-Chains MonteCarlo for 10^7^ generations sampling every 1000 generations. Convergence across runs was evaluated using two methods: a) the examination of the standard deviation of split frequencies (with acceptance values < 0.01); and b) by verification of parameter estimates in TRACER v1.6 [114], based on acceptable effective sample sizes (ESS values > 200). After checking for convergence, the first 25% of the generated trees were discarded as burn-in and the remaining 75% were kept to calculate posterior probabilities. In addition, we also estimated phylogenetic trees using maximum likelihood (ML) criteria, as implemented in RAXMLGUI 1.5b1 [115–117], using the GTRCAT model, and estimated nodal support via 1000 bootstrap iterations using the selected partition. We selected closely-related taxa as outgroups, as suggested by previous published studies: *Aulacorhynchus albivitta* [108], *Chlorospingus flavopectus phaeocephalus* [103], *Cardellina versicolor* [5], and *Thalurania ridgwayi* [96, 97].

We used NETWORK 4.6.1.1 (Fluxus Engineering, www.fluxus-engineering.com) to visualize the relationships among haplotypes by constructing networks using the median-joining algorithm [118, 119], assigning equal weights to all variable sites and an epsilon parameter with default values (ε = 0).

To explicitly test for phylogeographic structure in each taxon avoiding a priori criteria to delineate populations, we used a Bayesian model-based clustering algorithm, as implemented in GENELAND 4.0.3 [120, 121], which assigns samples to clusters (K) according to both geographical adjacency and genetic similarity through simulations with the Reversible Jump Markov Chain MonteCarlo (RJMCMC) algorithm. We varied the maximum number of expected clusters (K_max_) from 2 to 10, following possible sub structuring of populations within the main montane ranges in Mesoamerica. We performed 10 independent runs of 10,000 iterations each, a thinning value of 1000 and a 10% burn-in. Best results were selected according to highest posterior probabilities.

Genetic structure was assessed through hierarchical analysis of molecular variance (AMOVA) using pairwise differences, based on the number of clusters (K) obtained by GENELAND for each taxon. Following these results, samples of *Aulacorhynchus* were divided into four population groups (K = 4): 1) Sierra Madre del Sur (SMS) samples from the Mexican states Guerrero and Oaxaca, 2) eastern Mexico and north Central America (EMx-NCA), 3) Costa Rica and northern Panama (CR-NP), and 4) southern Panama (SP); for *Chlorospingus,* we obtained K = 6: 1) SMS (Guerrero-Oaxaca), 2) northern Chiapas (including the Chimalapas region in Oaxaca), 3) northeastern Oaxaca and Sierra Madre Oriental (SMO), 4) Los Tuxtlas Massif (TM), 5) northern Central America (NCA), and 6) southern Central America (SCA). Samples of *Cardellina* were divided into K = 4: 1) Trans Mexican Volcanic Belt (TMVB), 2) SMS, 3) Sierra Madre Occidental (SMOc), and 4) northern Central America (NCA-*C. versicolor*). Finally, the *Eupherusa-Thalurania* samples, where GENELAND clustering followed current species delimitation: 1) *E. cyanophrys* (Sierra de Miahuatlán, Oaxaca), 2) *E. poliocerca* (Guerrero-Oaxaca), 3) *E. eximia* (N Oaxaca-N Central America), 4) *E. nigriventris* (Costa Rica-Panama), and 5) *Thalurania rigdwayi* (Jalisco).

We also assessed genetic diversity of each population group within each species through the estimation of haplotype diversity (Hd), and nucleotide diversity (π). Genetic divergence between groups was measured using pairwise *F*_ST_ fixation index [122, 123], interpreting the results following guides in [90]. Significance of *F*_ST_ tests was assessed using 1000 permutations. To test for evidence of recent demographic changes in the selected species, we estimated historical demographic dynamics through the calculation of Fu’s F_S_ statistic [124] and Tajima’s D statistic [125] neutrality tests. Significance of these tests (*p* <  0.02 in the case of F_S_ statistic) was calculated through 1000 simulations. All analyses were conducted in ARLEQUIN 3.1 [126].

### Divergence time estimation

We used a Bayesian MCMC-based approach to calculate divergence times among haplogroups within each species independently using BEAST v1.8.4 [127]. For each selected taxon, we first tested whether our dataset fits either to a strict clock model or to a relaxed clock model. We performed selection tests through the stepping-stone method (SS [128];), as implemented in MRBAYES 3.2 [113]. Given our partitioning models, the mean marginal likelihood of a strict clock performed better than a relaxed clock model for all taxa (Additional file 2). Thus, chains were run using a UPGMA starting tree, under a strict clock with substitution models (according to results from PARTITIONFINDER) for 10^8^ generations sampling every 1000 steps, using a Yule speciation process [129, 130] with no topological constraints, and discarded the first 25% as burn-in. We used two approximations to convert branch lengths into time: (A) a body mass-corrected molecular clock rate of 0.0042 (min = 0.0011, max = 0.0158) subs/site/Myr [131], and (B) an uncorrelated lognormal relaxed clock substitution rate fixed at 0.01 average subs/site and SD = 0.003 [132–134] for each locus calculated in BEAST v1.8.4 [127]. Adjustment of the body mass-corrected and calculated clocks was evaluated using Bayes factors (BF), calculated from the marginal likelihoods from path sampling (PS) and stepping-stone (SS) methods in BEAST v1.8.4 [127, 135, 136]. Each marginal likelihood was estimated through 100 path steps with a Beta distribution (0.3, 1.0). We considered a 3 Log ml difference as strong evidence [137] against the null hypothesis (Additional file 2). We checked for stationarity using TRACER v1.6 [114]. Node ages are presented as mean heights and 95% credibility interval values with a posterior probability limit of 0.5 and resumed in a maximum clade credibility tree (MCCT). All of these were generated using TREEANNOTATOR v1.8.4 [127], and trees were visualized in FIGTREE v1.4.2 (http://tree.bio.ed.ac.uk/software/figtree/).

### Coalescent-based estimation of population history

We tested for different hypotheses of population divergence and admixture using an approximate Bayesian computation (ABC) approach in DIYABC 2.1.0 [138]. We conducted initial simulations using 15 competing evolutionary scenarios per species (Additional file 3). Evolutionary scenarios for each species were built considering results of the phylogenetic and GENELAND analyses, biogeographic diversification scenarios proposed in previous studies (see [68, 106, 109]), and geographical breaks invoked as responsible of diversification events. We followed recommendations in [50, 139, 140] for the assessment of appropriate priors, as well as to select the highly-informative summary statistics from the simulations resembling datasets similar to the empirical ones. When phylogenies suggested the existence of two genetic groups within the SMS (p. e. distinctive Guerrero and Oaxaca lineages, as in *Aulacorhynchus* and *Chlorospingus*), we contrasted the same scenarios considering those clades as different populations in the analyses. All competing scenarios were eliminated in successive rounds, where the preferred hypotheses were the ones with highest posterior probabilities.

From the selection process described above, we tested three final models for *Aulacorhynchus*, *Chlorospingus*, and *Cardellina*; and two for *Eupherusa-Thalurania* (Additional file 5). For each matrix analyzed, we simulated 1 × 10^6^ datasets and obtained summary statistics per scenario in each simulation. Based on rates reported previously for birds [141–145], we used an HKY mutation model with a uniform prior distribution, a mean mutation rate with a gamma distribution set to 2.0 × 10^− 8^ substitutions/site/year (min = 1.6 × 10^− 8^, max = 2.9 × 10^− 8^ substitutions/site/year) The posterior probabilities of competing scenarios were computed using a logistic regression on the 1% of simulated datasets closest to the observed data. The selected scenario was that with highest probability value and a non-overlapping 95% confidence interval. For the best supported scenario, we performed a model-checking procedure using a Principal Components Analysis (PCA) on test statistics to visualize the fit between simulated and observed datasets. Confidence on the chosen scenario was assessed though the calculation of type I and type II error rate, from 500 simulated pseudo-observed datasets (PODs) generated with the data of the best-supported scenario [146, 147]. Point estimates for demographic and temporal parameters were obtained by local linear regression on the 1% of simulations closest to the observed dataset for the best-supported scenario [138]. Divergence time obtained from DIYABC output was transformed assuming a conservative two-year generation time, which has been previously used in similar species groups [66, 148].

### Test of simultaneous divergence

We tested whether genetic differentiation occurred simultaneously between the main geographical barriers in the analyzed clusters by performing a hierarchical approximate Bayesian computation (hABC) analysis as implemented in MSBAYES [4]. Populations inhabiting the montane regions throughout the study area showed similar patterns of genetic structuring even when they are not closely related, we may therefore expect inter-specific simultaneous divergence among regions if the processes driving diversification are common. We performed hABC analyses in 15 population pairs that spanned the same putative barriers to gene flow: between populations within the SMS in southwestern Mexico; between populations of the SMS and eastern populations across a lowland barrier in Oaxaca’s Central Valleys, and between populations spanning the Nicaraguan Depression in Central America (Fig. 1).

We used jModelTest [149, 150] to estimate a transition-transversion rate for each population pair, as required by MSBAYES. The analyses involved the estimation of a vector of summary statistics from the sequences utilized; afterwards 1 × 10^6^ data sets were simulated under the specified multi-taxon model using prior distribution for the demographic parameters. The prior of the maximum possible number of divergence events (Ψ) was set to be equal to the number of lineage pairs tested. For the last stage of the MSBAYES analyses, we used the acceptance/rejection algorithm to approximate the posterior distribution for the hyper parameters that characterize the degree of variability of demographic and temporal parameters given the empirical data (e. g., τ, Ψ, Ω). We used Ω estimates to evaluate the support for each hypothesis: synchronous diversification is expected when Ω ≤ 0.01; asynchronous diversification if Ω > 0.01 [4, 18, 151, 152]. To estimate mean divergence times, we converted model-averaged *E*(τ) estimates (provided in MSBAYES as coalescent units of 4N_AVE_ generations) to absolute time (T_div_), using the equation T_div_ *= E*(τ) × (θ_AVE_/μ) × g, where g is the generation time in years, and θ_AVE_ is the mean of the upper θ prior [151], and μ is the mutation rate. We used a mean mutation rate of 0.0042 substitutions/site/Myr prior [131], and an average generation time of 2 years [66, 148].

## Supplementary information

**Additional file 1:** Genbank accession numbers. Samples obtained for the realization of analyses in this study.

**Additional file 2:** Marginal likelihoods for sequence evolution. Marginal likelihoods estimation for sequence fit to a strict or relaxed clock and comparison of evolutionary rate assumptions through Bayes factors (BFs).

**Additional file 3: **Hypothetical scenarios of diversification of 4 bird taxa in Mesoamerica. Scenarios tested with ABC for *Aulacorhynchus, Chlorospingus, Cardellina, and Eupherusa-Thalurania* with confidence scenario choice values (Type I and II errors).

**Additional file 4: **Posterior parameter estimates for the best supported scenarios. Population and divergence time estimates obtained with ABC for *Aulacorhynchus, Chlorospingus, Cardellina, and Eupherusa-Thalurania. (DOCX 33 kb)*

**Additional file 5: **Final tested hypothetical scenarios of diversification of 4 bird taxa in Mesoamerica. Competing demographic scenarios of *Aulacorhynchus, Chlorospingus, Cardellina, and Eupherusa-Thalurania* with posterior probabilities and model checking.

## Data Availability

All of the data generated or analyzed during this study are included in this published article [and its supplementary information files].

## Abbreviations

CR-NP: Costa Rica and northern Panama
EMx-NCA: Eastern Mexico and north Central America
N Chiapas: Northern Chiapas
NCA: Northern Central America
SCA: Southern Central America
SMO: Northeastern Oaxaca and Sierra Madre Oriental
SMOc: Sierra Madre Occidental
SMS: Sierra Madre del Sur
SP: Southern Panama
TM: Tuxtlas Massif
TMVB: Trans-Mexican Volcanic Belt
