# Supplementary material for: The geography of evolutionary divergence in the highly endemic avifauna from the Sierra Madre del Sur, Mexico
Source: BMC Evol Biol. 2019 Dec 30;19:237. doi: 10.1186/s12862-019-1564-3 (PMC6937948; doi:10.1186/s12862-019-1564-3)
Supplement: Supplementary file 1 — Additional file 1: Genbank accession numbers. Samples obtained for the realization of analyses in this study. [file 12862_2019_1564_MOESM1_ESM.docx]

**The geography of evolutionary divergence in the highly endemic avifauna from the Sierra Madre del Sur, Mexico**

ALBERTO ROCHA-MÉNDEZ, LUIS A. SÁNCHEZ-GONZÁLEZ, CLEMENTINA GONZÁLEZ, & ADOLFO G. NAVARRO-SIGÜENZA

**Supporting Information**

Table 1. GenBank accession numbers of samples analyzed in this study.

| Species | ND2 GenBank | ND3 GenBank | Cytb GenBank | COII ATPase 6 & 8 GenBank | Longitude | Latitude | Voucher |
| --- | --- | --- | --- | --- | --- | --- | --- |
| *A. prasinus* | EU285742 | EU285803 | EU285681 |  | -98.955 | 21.075 | SLP139 |
| *A. prasinus* | EU285743 | EU285804 | EU285682 |  | -96.96 | 18.81 | NAR29 |
| *A. prasinus* | EU285744 | EU285805 | EU285683 |  | -96.72 | 18.15 | OMVP1071 |
| *A. prasinus* | EU285745 | EU285806 | EU285684 |  | -97.58 | 20.03 | PUE154 |
| *A. prasinus* | EU285746 | EU285807 | EU285685 |  | -99.065 | 21.273 | QRO324 |
| *A. prasinus* | EU285748 | EU285809 | EU285687 |  | -94.809 | 18.363 | TUX02 |
| *A. prasinus* | EU285747 | EU285808 | EU285686 |  | -94.809 | 18.363 | TUX01 |
| *A. prasinus* | EU285749 | EU285810 | EU285688 |  | -94.809 | 18.363 | TUX03 |
| *A. prasinus* | EU285732 | EU285793 | EU285671 |  | -99.748 | 17.626 | CAON78 |
| *A. prasinus* | EU285733 | EU285794 | EU285672 |  | -100.913 | 17.625 | CAON147 |
| *A. prasinus* | EU285734 | EU285795 | EU285673 |  | -100.913 | 17.625 | CAON149 |
| *A. prasinus* | EU285735 | EU285796 | EU285674 |  | -97.84 | 16.84 | OMVP697 |
| *A. prasinus* | EU285738 | EU295799 | EU285677 |  | -96.42 | 15.93 | CONA205 |
| *A. prasinus* | EU285739 | EU285800 | EU285678 |  | -96.42 | 15.93 | CONA206 |
| *A. prasinus* | EU285736 | EU285797 | EU285675 |  | -97.84 | 16.84 | OMVP705 |
| *A. prasinus* | EU285737 | EU285798 | EU285676 |  | -97.84 | 16.84 | OMVP708 |
| *A. prasinus* | EU285741 | EU285802 | EU285680 |  | -99.036 | 21.086 | BMM899 |
| *A. prasinus* | EU285740 | EU285801 | EU285679 |  | -99.036 | 21.086 | BMM898 |
| *A. prasinus* | EU285752 | EU285811 | EU285691 |  | -90.708 | 14.49 | DHB4450 |
| *A. prasinus* | EU285753 | EU285812 | EU285692 |  | -88.2 | 13.762 | EAGT38 |
| *A. prasinus* | EU285755 | EU285814 | EU285694 |  | -88.271 | 13.434 | PUE02 |
| *A. prasinus* | EU285757 | EU285816 | EU285696 |  | -85.887 | 12.94 | DAB13140 |
| *A. prasinus* | EU285758 | EU285817 | EU285697 |  | -85.887 | 12.94 | DAB1367 |
| *A. prasinus* | EU285759 | EU285818 | EU285698 |  | -85.887 | 12.94 | DAB1368 |
| *A. prasinus* | EU285760 | EU285819 | EU285699 |  | -83.911 | 9.776 | UCR1211 |
| *A. prasinus* | EU285761 | EU285820 | EU285700 |  | -84.716 | 10.25 | UCR3965 |
| *A. prasinus* | EU285763 | EU285822 | EU285702 |  | -82.405 | 8.805 | LSUMZ26464 |
| *A. prasinus* | EU285764 | EU285823 | EU285703 |  | -82.405 | 8.805 | LSUMZ26403 |
| *A. prasinus* | EU285765 | EU285824 | EU285704 |  | -78.15 | 8.4166 | LSUMZ-1373 |
| *A. albivitta* | EU285784 | EU285842 | EU285723 |  | -72.57 | 10.364 | COP81127 |
| *A. albivitta* | EU285785 | EU285843 | EU285724 |  | -72.57 | 10.364 | COP81128 |
| *A. albivitta* | EU285786 | EU285844 | EU285725 |  | -72.57 | 10.364 | COP81129 |
| *C. flavopectus* |  |  |  | AY609288 | -94.653 | 17.054 | MZFC12084 |
| *C. flavopectus* |  |  |  | AY609282 | -96.846 | 18.17 | MZFC11585 |
| *C. flavopectus* |  |  |  | AY609295 | -97.795 | 17.025 | MZFC11579 |
| *C. flavopectus* |  |  |  | AY609281 | -96.996 | 18.165 | MZFC12940 |
| *C. flavopectus* |  |  |  | AY609294 | -97.8 | 17.018 | MZFC12810 |
| *C. flavopectus* |  |  |  | AY609290 | -94.683 | 16.908 | MZFCB18090 |
| *C. flavopectus* |  |  |  | AY609289 | -94.683 | 16.908 | MZFCB18089 |
| *C. flavopectus* |  |  |  | AY609283 | -95.99 | 17.307 | MZFCMXJ511 |
| *C. flavopectus* |  |  |  | AY609293 | -100.218 | 17.486 | MZFCMX1437 |
| *C. flavopectus* |  |  |  | AY609280 | -97.15 | 19.483 | IECO065 |
| *C. flavopectus* |  |  |  | AY609285 | -95.2 | 18.558 | MZFCMX1078 |
| *C. flavopectus* |  |  |  | AY609284 | -95.2 | 18.558 | MX1080Tuxtla |
| *C. flavopectus* |  |  |  | EU594960 | -98.314 | 20.466 | MZFC16209 |
| *C. flavopectus* |  |  |  | EU594961 | -98.314 | 20.466 | MZFC16236 |
| *C. flavopectus* |  |  |  | EU594965 | -94.9 | 18.316 | FM393777 |
| *C. flavopectus* |  |  |  | EU594966 | -94.9 | 18.316 | FM393778 |
| *C. flavopectus* |  |  |  | EU594967 | -94.9 | 18.316 | FM393779 |
| *C. flavopectus* |  |  |  | EU594968 | -94.9 | 18.316 | FM393876 |
| *C. flavopectus* |  |  |  | EU594969 | -100.218 | 17.486 | FM394049 |
| *C. flavopectus* |  |  |  | EU594970 | -100.218 | 17.486 | FM394050 |
| *C. flavopectus* |  |  |  | EU594971 | -100.218 | 17.486 | FM394051 |
| *C. flavopectus* |  |  |  | EU594972 | -100.218 | 17.486 | FM394052 |
| *C. flavopectus* |  |  |  | EU594973 | -100.218 | 17.486 | FM394053 |
| *C. flavopectus* |  |  |  | EU594974 | -95.99 | 17.307 | FM346811 |
| *C. flavopectus* |  |  |  | EU594975 | -95.99 | 17.307 | FM346812 |
| *C. flavopectus* |  |  |  | EU594976 | -95.99 | 17.307 | FM346813 |
| *C. flavopectus* |  |  |  | EU594977 | -95.99 | 17.307 | FM393783 |
| *C. flavopectus* |  |  |  | EU594978 | -95.99 | 17.307 | FM393784 |
| *C. flavopectus* |  |  |  | EU594979 | -95.99 | 17.307 | FM393785 |
| *C. flavopectus* |  |  |  | EU594980 | -96.84 | 18.13 | MZFC12752 |
| *C. flavopectus* |  |  |  | EU594981 | -96.84 | 18.16 | MZFC11588 |
| *C. flavopectus* |  |  |  | EU594982 | -96.84 | 18.16 | MZFC12748 |
| *C. flavopectus* |  |  |  | EU594953 | -97.516 | 20.091 | MZFC11303 |
| *C. flavopectus* |  |  |  | EU594954 | -97.583 | 19.883 | BMJK04712 |
| *C. flavopectus* |  |  |  | EU594955 | -97.583 | 19.883 | BMGMS1414 |
| *C. flavopectus* |  |  |  | EU594956 | -99.312 | 21.776 | MZFC13112 |
| *C. flavopectus* |  |  |  | EU594958 | -98.618 | 20.626 | MZFC10630 |
| *C. flavopectus* |  |  |  | AY609287 | -92.083 | 17.183 | MZFC9573 |
| *C. flavopectus* |  |  |  | EU594983 | -90.708 | 14.49 | BMDHB4452 |
| *C. flavopectus* |  |  |  | EU594984 | -90.708 | 14.49 | BMGAV2384 |
| *C. flavopectus* |  |  |  | AY609297 | -89.123 | 14.381 | KU4885 |
| *C. flavopectus* |  |  |  | AY609296 | -89.123 | 14.381 | KU4892 |
| *C. flavopectus* |  |  |  | EU594987 | -89.403 | 14.362 | KU9424 |
| *C. flavopectus* |  |  |  | EU594988 | -89.403 | 14.362 | KU9425 |
| *C. flavopectus* |  |  |  | EU594989 | -89.05 | 14.866 | BMGAV1537 |
| *C. flavopectus* |  |  |  | EU594990 | -89.05 | 14.866 | BMJK9974 |
| *C. flavopectus* |  |  |  | EU594994 | -85.845 | 12.94 | BMDAB1291 |
| *C. flavopectus* |  |  |  | EU594995 | -85.845 | 12.94 | UWBM56129 |
| *C. flavopectus* |  |  |  | EU594998 | -84.17 | 10.255 | LSUMNS16001 |
| *C. flavopectus* |  |  |  | EU594999 | -84.17 | 10.255 | LSUMNS16005 |
| *C. flavopectus* |  |  |  | EU595000 | -83.813 | 9.77 | LSUMNS35752 |
| *C. flavopectus* |  |  |  | EU595001 | -82.42 | 9.34 | NMNHB2020 |
| *C. flavopectus* |  |  |  | EU595002 | -82.42 | 9.34 | NMNHB2021 |
| *C. flavopectus phaeocephalus* |  |  |  | EU595005 | -78.13 | -2.366 | UCLA00N426 |
| *C. flavopectus phaeocephalus* |  |  |  | EU595006 | -78.13 | -2.366 | UCLA00N41 |
| *C. rubra* | JQ282424 |  | JQ282552 | JQ282620 | -102.319 | 19.41 | FMNH394171 |
| *C. rubra* | JQ282425 |  | GU932413 | JQ282621 | -102.319 | 19.41 | FMNH394172 |
| *C. rubra* | JQ282458 |  | JQ282483 | JQ282585 | -95.8 | 16.6 | OMVP0373 |
| *C. rubra* | JQ282441 |  | JQ282484 | JQ282579 | -97.61 | 17.15 | OMVP138 |
| *C. rubra* | JQ282459 |  | JQ282485 | JQ282580 | -95.8 | 16.6 | OMVP0357 |
| *C. rubra* | JQ282444 |  | JQ282486 | JQ282578 | -95.8 | 16.6 | OMVP0358 |
| *C. rubra* | JQ282442 |  | JQ282487 | JQ282577 | -95.8 | 16.6 | OMVP0314 |
| *C. rubra* | JQ282460 |  | JQ282488 | JQ282581 | -97.61 | 17.15 | OMVP124 |
| *C. rubra* | JQ282443 |  | JQ282489 | JQ282584 | -95.8 | 16.6 | OMVP0415 |
| *C. rubra* | JQ282461 |  | JQ282490 | JQ282586 | -97.61 | 17.15 | OMVP0094 |
| *C. rubra* | JQ282445 |  | JQ282491 | JQ282583 | -96.85 | 17.64 | OMVP0787 |
| *C. rubra* | JQ282440 |  | JQ282492 | JQ282582 | -97.61 | 17.15 | MZFC119 |
| *C. rubra* | JQ282447 |  | JQ282493 | JQ282587 | -105.87 | 23.59 | RCF2800 |
| *C. rubra* | jq282446 |  | JQ282494 | JQ282588 | -105.87 | 23.59 | VGR1054 |
| *C. rubra* | JQ282437 |  | JQ282496 | JQ282630 | -103.62 | 19.62 | ENT104 |
| *C. rubra* | JQ282438 |  | JQ282497 | JQ282591 | -103.62 | 19.62 | ENT105 |
| *C. rubra* | JQ282453 |  | JQ282498 | JQ282612 | -99.29 | 18.27 | FD117 |
| *C. rubra* | JQ282449 |  | JQ282499 | JQ282632 | -99.33 | 19.29 | BIODF26 |
| *C. rubra* | JQ282450 |  | JQ282500 | JQ282635 | -99.33 | 19.29 | BIODF027 |
| *C. rubra* | JQ282451 |  | JQ282501 | JQ282636 | -99.33 | 19.29 | BIODF28 |
| *C. rubra* | JQ282462 |  | JQ282502 | JQ282637 | -99.27 | 19.28 | BIODF29 |
| *C. rubra* | JQ282472 |  | JQ282507 | JQ282640 | -98.59 | 19.1 | ENT09076 |
| *C. rubra* | JQ282470 |  | JQ282508 | JQ282641 | -98.59 | 19.1 | ENT09078 |
| *C. rubra* | JQ282466 |  | JQ282509 | JQ282590 | -98.59 | 19.1 | SEK115 |
| *C. rubra* | JQ282474 |  | JQ282510 | JQ282589 | -98.59 | 19.1 | SEK116 |
| *C. rubra* | JQ282464 |  | JQ282511 | JQ282625 | -99.29 | 18.96 | MT409 |
| *C. rubra* | JQ282454 |  | JQ282512 | JQ282596 | -99.31 | 19.04 | jk04-016 |
| *C. rubra* | JQ282468 |  | JQ282513 | JQ282594 | -99.96 | 17.81 | jk04-156 |
| *C. rubra* | JQ282455 |  | JQ282514 | JQ282619 | -99.96 | 17.81 | jk04-167 |
| *C. rubra* | JQ282479 |  | JQ282515 | JQ282611 | -99.96 | 17.81 | jk04-171 |
| *C. rubra* | JQ282456 |  | JQ282516 | JQ282593 | -99.96 | 17.81 | jk04-206 |
| *C. rubra* | JQ282457 |  | JQ282517 | JQ282617 | -99.96 | 17.81 | jk04-207 |
| *C. rubra* | JQ282402 |  | JQ282519 | JQ282570 | -102.26 | 19.43 | mm520 |
| *C. rubra* | JQ282405 |  | JQ282520 | JQ282574 | -102.26 | 19.43 | mm513 |
| *C. rubra* | JQ282403 |  | JQ282521 | JQ282571 | -102.26 | 19.43 | mm494 |
| *C. rubra* | JQ282404 |  | JQ282522 | JQ282573 | -102.24 | 19.42 | mm445 |
| *C. rubra* | JQ282478 |  | JQ282523 | JQ282568 | -99.19 | 19.08 | jmd474 |
| *C. rubra* | JQ282427 |  | JQ282524 | JQ282566 | -99.19 | 19.08 | jmd457 |
| *C. rubra* | JQ282426 |  | JQ282525 | JQ282567 | -99.19 | 19.08 | jmd456 |
| *C. rubra* | JQ282423 |  | JQ282526 | JQ282600 | -99.36 | 19.13 | jk08-322 |
| *C. rubra* | JQ282415 |  | JQ282527 | JQ282623 | -102.26 | 19.43 | jk06-509 |
| *C. rubra* | JQ282413 |  | JQ282528 | JQ282610 | -102.24 | 19.43 | jk06-508 |
| *C. rubra* | JQ282420 |  | JQ282529 | JQ282626 | -102.24 | 19.43 | jk06-450 |
| *C. rubra* | JQ282421 |  | JQ282531 | JQ282615 | -97.57 | 19.83 | jk04-754 |
| *C. rubra* | JQ282418 |  | JQ282532 | JQ282602 | -97.57 | 19.83 | jk04-753 |
| *C. rubra* | JQ282419 |  | JQ282533 | JQ282629 | -99.084 | 17.61 | jk04-353 |
| *C. rubra* | JQ282417 |  | JQ282534 | JQ282597 | -99.084 | 17.61 | jk04-351 |
| *C. rubra* | JQ282414 |  | JQ282535 | JQ282601 | -99.084 | 17.61 | jk04-349 |
| *C. rubra* | JQ282439 |  | JQ282536 | JQ282616 | -99.84 | 17.61 | jk04-080 |
| *C. rubra* | JQ282416 |  | JQ282537 | JQ282618 | -99.84 | 17.61 | jk04-079 |
| *C. rubra* | JQ282410 |  | JQ282542 | JQ282592 | -99.19 | 19.08 | gms1457 |
| *C. rubra* | JQ282412 |  | JQ282543 | JQ282598 | -99.19 | 19.08 | gms1455 |
| *C. rubra* | JQ282475 |  | JQ282551 | JQ282631 | -99.25 | 19.24 | BIODF05 |
| *C. rubra* | JQ282401 |  | JQ282556 | JQ282575 | -102.26 | 19.43 | mm500 |
| *C. rubra* | JQ282406 |  | JQ282557 | JQ282572 | -102.24 | 19.38 | mm451 |
| *C. rubra* | JQ282428 |  | JQ282558 | JQ282569 | -99.85 | 17.6 | jmd424 |
| *C. rubra* | JQ282430 |  | JQ282559 | JQ282609 | -99.19 | 19.08 | gms1516 |
| *C. rubra* | JQ282481 |  | JQ282562 | JQ282643 | -99.54 | 17.38 | PBSMS05 |
| *C. versicolor* | JQ282433 |  | JQ282538 | JQ282603 | -91.49 | 14.82 | jk02-096 |
| *C. versicolor* | JQ282431 |  | JQ282539 | JQ282605 | -91.49 | 14.82 | jk02-050 |
| *C. versicolor* | JQ282435 |  | JQ282546 | JQ282604 | -91.49 | 14.82 | gav2370 |
| *C. versicolor* | JQ282432 |  | JQ282547 | JQ282606 | -91.49 | 14.82 | gav2349 |
| *C. versicolor* | JQ282434 |  | JQ282560 | JQ282608 | -91.91 | 15.14 | 03N1073 |
| *E. nigriventris* |  |  |  |  | -84.16 | 10.27 | B16051 |
| *E. nigriventris* | EU042553 |  |  |  | -84.16 | 10.27 | B16055 |
| *E. eximia* |  |  |  |  | -94.583 | 17.066 | CHIMA161 |
| *E. eximia* |  |  |  |  | -94.583 | 17.066 | CHIMA181 |
| *E. eximia* |  |  |  |  | -94.583 | 17.066 | CHIMA318 |
| *E. eximia* |  |  |  |  | -94.583 | 17.066 | CHIMA378 |
| *E. eximia* |  |  |  |  | -94.658 | 17.02 | OMVP558 |
| *E. eximia* |  |  |  |  | -94.583 | 17.066 | OMVP505 |
| *E. eximia* | EU042552 |  |  |  | -94.655 | 17.055 | B21998 |
| *E. cyanoprhys* |  |  |  |  | -96.427 | 15.934 | CONACYT04202 |
| *E. cyanoprhys* |  |  |  |  | -96.427 | 15.934 | CONACYT04231 |
| *E. cyanoprhys* |  |  |  |  | -96.427 | 15.934 | CONACYT04240 |
| *E. cyanoprhys* |  |  |  |  | -96.427 | 15.934 | PLU030 |
| *E. cyanoprhys* | KJ602243 |  |  |  | -96.427 | 15.934 | B21997 |
| *E. poliocerca* |  |  |  |  | -100.2 | 17.44 | ATO031 |
| *E. poliocerca* |  |  |  |  | -100.2 | 17.44 | ATO034 |
| *E. poliocerca* |  |  |  |  | -100.2 | 17.44 | ATO043 |
| *E. poliocerca* |  |  |  |  | -100.067 | 17.065 | BEHB200 |
| *E. poliocerca* |  |  |  |  | -100.067 | 17.065 | BEHB215 |
| *E. poliocerca* |  |  |  |  | -97.94 | 17.04 | OMVP655 |
| *E. poliocerca* |  |  |  |  | -97.95 | 17.08 | OMVP657 |
| *E. poliocerca* |  |  |  |  | -100.2 | 17.416 | SIT043 |
| *E. poliocerca* |  |  |  |  | -100.2 | 17.416 | SIT049 |
| *E. poliocerca* | KJ602244 |  |  |  | -100.2 | 17.4 | B21999 |
| *T. ridwayi* | KJ602349 |  |  |  | -104.395 | 19.688 | FMNHMEX312 |
| *T. ridwayi* | KJ602348 |  |  |  | -104.395 | 19.688 | FMNHMEX305 |
| *T. ridwayi* | KJ602347 |  |  |  | -104.395 | 19.688 | FMNHMEX225 |
