# Supplementary material for: The geography of evolutionary divergence in the highly endemic avifauna from the Sierra Madre del Sur, Mexico
Source: BMC Evol Biol. 2019 Dec 30;19:237. doi: 10.1186/s12862-019-1564-3 (PMC6937948; doi:10.1186/s12862-019-1564-3)
Supplement: Supplementary file 2 — Additional file 2: Marginal likelihoods for sequence evolution. Marginal likelihoods estimation for sequence fit to a strict or relaxed clock and comparison of evolutionary rate assumptions through Bayes factors (BFs). [file 12862_2019_1564_MOESM2_ESM.docx]

**The geography of evolutionary divergence in the highly endemic avifauna from the Sierra Madre del Sur, Mexico**

ALBERTO ROCHA-MÉNDEZ, LUIS A. SÁNCHEZ-GONZÁLEZ, CLEMENTINA GONZÁLEZ, & ADOLFO G. NAVARRO-SIGÜENZA

**Supporting Information**

Table 2. Marginal likelihood estimates to a relaxed and strict clock for sequence evolution, estimated through the stepping-stone method (Xie et al., 2011), showing that the strict clock performed better than a relaxed clock for all species.

| Dataset | Relaxed clock’s mean marginal likelihood (-Ln) | Strict clock’s mean marginal likelihood (-Ln) |
| --- | --- | --- |
| *Aulacorhynchus* | 2859.22 | 2765.16 |
| *Chlorospingus* | 2727.45 | 2664.42 |
| *Cardellina* | 4766.77 | 4427.2 |
| *Eupherusa* | 2298.05 | 2228.31 |

Table 3. Marginal likelihood estimation (Log ml) calculated in BEAST using path sampling (PS) and stepping-stone (SS) methods under two evolutionary rate assumptions: calculated and body mass-corrected. Shown Bayes factors (BFs) are the result of comparisons made between Log mls obtained through SS. Asterisks (*) after (log_10_) BF indicate the following according to Raftery (1996): * low support of the hypothesis, ** positive support for the model, and *** very strong evidence favoring the hypothesis with higher log(ml).

| Dataset | Evolution Rate | Log ml (PS) | Log ml (SS) | BFs |
| --- | --- | --- | --- | --- |
| *Aulacorhynchus* | Calculated | -4126.871875 | -4127.724235 | -392.41344 |
|  | Body mass-corrected | -3831.222778 | -3735.310795 | 392.41344*** |
| *Chlorospingus* | Calculated | -2730.394245 | -2731.516758 | -2.726148 |
|  | Body mass-corrected | -2728.641854 | -2728.79061 | 2.726148* |
| *Cardellina* | Calculated | -2556.849255 | -2557.792954 | -2.095804 |
|  | Body mass-corrected | -2554.271126 | -2555.69715 | 2.095804* |
| *Eupherusa* | Calculated | -2215.229914 | -2216.60914 | -3.12914 |
|  | Body mass-corrected | -2212.859479 | -2213.48 | 3.12914** |
