# Supplementary material for: The geography of evolutionary divergence in the highly endemic avifauna from the Sierra Madre del Sur, Mexico
Source: BMC Evol Biol. 2019 Dec 30;19:237. doi: 10.1186/s12862-019-1564-3 (PMC6937948; doi:10.1186/s12862-019-1564-3)
Supplement: Supplementary file 3 — Additional file 3: Hypothetical scenarios of diversification of 4 bird taxa in Mesoamerica. Scenarios tested with ABC for Aulacorhynchus, Chlorospingus, Cardellina, and Eupherusa-Thalurania with confidence scenario choice values (Type I and II errors). [file 12862_2019_1564_MOESM3_ESM.docx]

**The geography of evolutionary divergence in the highly endemic avifauna from the Sierra Madre del Sur, Mexico**

ALBERTO ROCHA-MÉNDEZ, LUIS A. SÁNCHEZ-GONZÁLEZ, CLEMENTINA GONZÁLEZ, & ADOLFO G. NAVARRO-SIGÜENZA

**Supporting Information**

*Hypothetical scenarios of diversification of 4 bird taxa in Mesoamerica.*

Table 4. Confidence in scenario choice (Type I and II errors) selected as the real scenario by logistic approach for each of the 4 taxa analyzed. Scenario evaluation includes cases where population differentiation was observed within the SMS highlands.

| **Evaluated Scenario** | **Type I** | **Type II** |
| --- | --- | --- |
| *Aulacorhynchus* (four population groups) | 0.285 | 0.231 |
| *Aulacorhynchus* (five populations groups) | 0.45 | 0.4 |
| *Chlorospingus* (six population groups) | 0.357 | 0.44 |
| *Chlorospingus* (seven population groups) | 0.282 | 0.43 |
| *Cardellina* | 0.367 | 0.137 |
| *Eupherusa* (including *T. ridgwayi*) | 0.212 | 0.288 |
| *Eupherusa* (excluding *T. ridgwayi*) | 0.319 | 0.219 |

***Aulacorhynchus***

Populations:

1: EMx-NCA (Eastern Mexico and northern Central America)

2: SMS (Guerrero & Oaxaca)

3: CR-NP (Costa Rica and northern Panama)

4: SP (southern Panama)

Tested scenarios: *H0 scenario, **selected real scenario.

1. Divergence between SMS and EMx-NCA from an ancestral population and succesive founder events EMx-NCA > CR-NP > SP.
2. Divergence between CR-NP and EMx-NCA from an ancestral population at t4. SMS population originates from EMx-NCA, and SP originates from CR-NP at t2.
3. Succesive founder events from populations SP > CR-NP > EMx-NCA > SMS.
4. Succesive founder events from populations SMS > EMx-NCA > CR-NP > SP at t4, t3, and t1 respectively.
5. Divergence of SP and EMx-NCA populations from an ancestral population at t4. At t2 simultaneous divergence of CR-NP and SMS populations.
6. Divergence between CR-NP and SP populations at t4 from an ancestral population. EMx-NCA population originates from CR-NP at t3. At t2 occurs an admixture event between EMx-NCA and CR-NP that originates SMS population.
7. *Simultaneous divergence of four *Aulacorhynchus* lineages at t4 from an ancestral popualtion.
8. Divergence of SMS population and a hypothetical ancestral population at 4. EMx-NCA population diverges at t3, and at t2 CR-NP and SP diverge from an hypothetical ancestral population.
9. Diversification between two hypothetical ancestral populations at t4. At t2 ancestral population south to the Nicaragua Depression diverges originating CR-NP and SP. Simultaneously EMx-NCA and SMS diverge.
10. Divergence of SP population and a hypothetical ancestral population at t4. CR-NP population diverges at t3, and at t2 EMx-NCA and SMS diverge from an hypothetical ancestral population.
11. **Divergence of an hypothetical ancestral population and SP at t4. CR-NP population diverges at t3, and at t2 EMx-NCA and SMS diverge from an hypothetical ancestral population.
12. Divergence between an hypothetical ancestral population and SP at t4. Simultaneous divergence of EMx-NCA, SMS, and CR-NP populations from an hypothetical ancestral population at t2.
13. Divergence at t4 from SP and a hypothetical ancestral population. Divergence of EMx-NCA and CR-NP from an hypothetical ancestral population at t3. At t2 admixture between EMx-NCA and CR-NP originates SMS population.
14. Divergence of SMS population at t4. Divergence from an ancestral population into EMx-NCA and an hypothetical ancestral population at t3. Divergence from the hypothetical ancestral population into CR-NP and SP populations.
15. Divergence of EMx-NCA and SP populations at t4. At t2 CR-NP population diverges from SP. Simultaneously SMS population diverges from EMx-NCA.


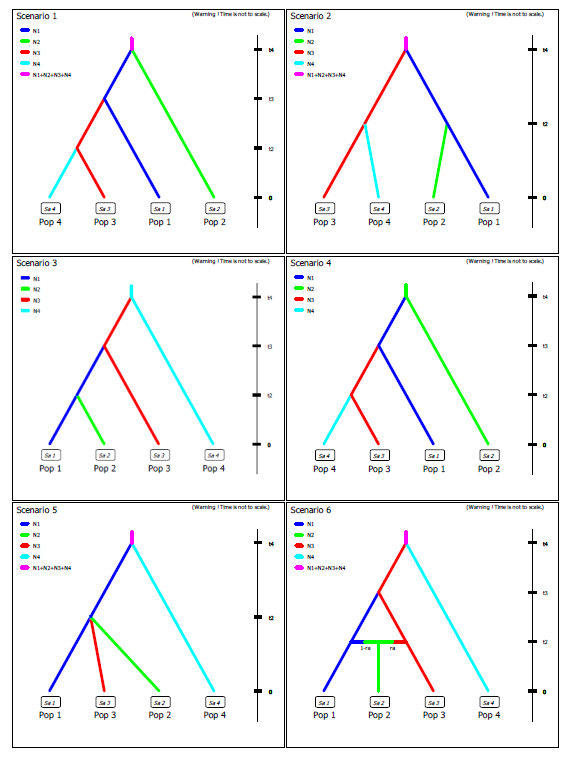


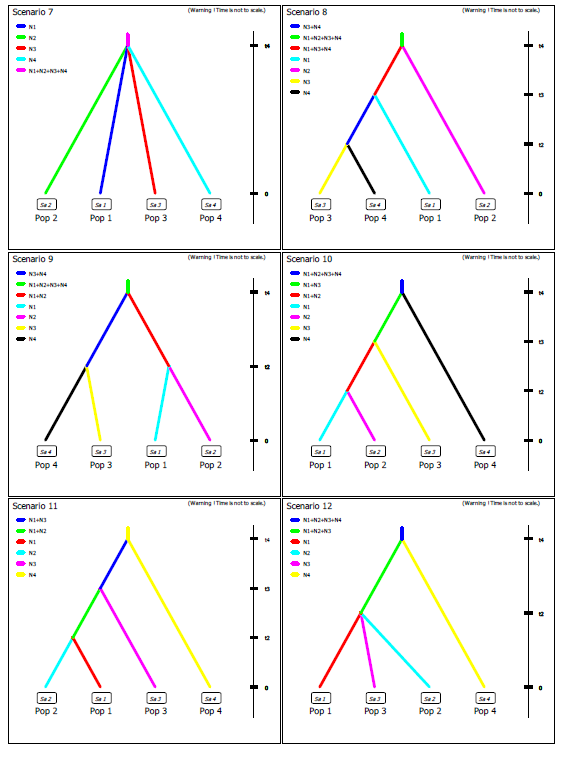


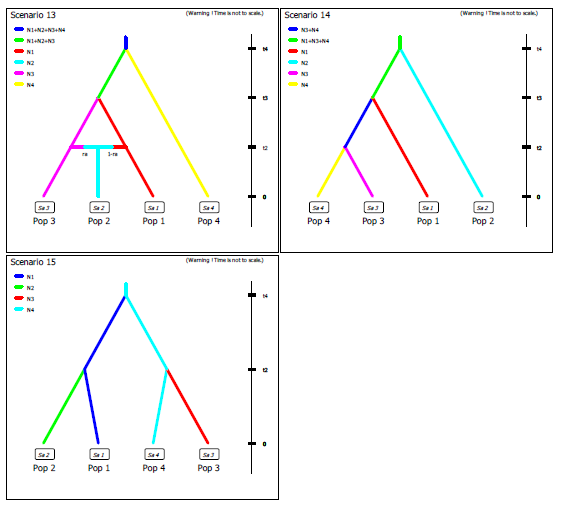


Figure 1. Scenarios tested with ABC for *Aulacorhynchus*. Numbers for each scenario correspond to the hypothesis in tested scenarios.

***Chlorospingus***

Populations:

1: TM (Los Tuxtlas Massif)

2: SMS (Guerrero & Oaxaca)

3: SMO (Sierra Madre Oriental and northeastern Oaxaca)

4: NChiapas (northern Chiapas and Chimalapas of Oaxaca)

5: NCA (northern Central America)

6: SCA (southern Central America)

Tested scenarios: *H0 scenario, **selected real scenario.

1. **Divergence between two hypothetical ancestral populations at t6. One lineage represents the ancestor of Central American populations. This lineage splits at t2 originating NCA and SCA populations. The other lineage represents the ancestor of all Mexican populations which has an east to west diversification.
2. Divergence of TM and NCA from an ancestral population at t6. Colonization from NCA population to SCA at t2. Westward expansion and divergence of populations.
3. Divergence of two hypothetical ancestral populations at t6. Divergence of NCA and SCA population at t2. Succesive colonization events TM > SMO > SMS > NChiapas at t5, t4, t3, and t2 respectively.
4. Divergence of SMS and SMO from an ancestral population at t6. Succesive colonization events SMO > TM > NChiapas > NCA > SCA at t5, t4, t3, and t2 respectively.
5. Divergence from an ancestral population of TM and NChiapas populations. Colonization events in Mexico with an east to west fashion TM > SMO > SMS. Colonization from NChiapas to Central America NChiapas > NCA > SCA.
6. Divergence of SMS and SCA from an ancestral population. Colonization events in Mexico follow a west to east fashion SMS > SMO > TM. Colonization from Central America to soouthern Mexico SCA > NCA > NChiapas.
7. Succesive colonization events following a south to north orientation. SCA > NCA > NChiapas > TM > SMO > SMS.
8. Divergence of NCA and SCA from an ancestral population. Colonization from NCA to northern Mesoamerica starts at t5, NCA > NChiapas > TM > SMO > SMS.
9. Divergence of SCA and an hypothetical ancestral population at t6. Northward expansion and splitting of populations.
10. *Simultaneous divergence from all Chlorospingus populations in Mesoamerica from an hypothetical ancestral popualtion at t6.
11. Divergence of two hypothetical ancestral populations. One at the east of the Isthmus of Tehuantepec (IT) which splits at t3 into NChiapas and NCA+SCA populations. The other ancestral population is west to the IT and splits at t3 into TM and SMS+SMO. At t2 NCA splits from SCA, and SMO from SMS.
12. Divergence of SMS and an hypothetical ancestral lineage of TM+SMO+NChiapas+NCA+SCA. Southward population expansion and diversification.
13. Divergence of an hypothetical ancestral lineage and SCA at t6. At t2 simultaneous diversification of NCA, NChiapas, TM, SMO and SMS populations.
14. Divergence of SCA and NCA populations from an ancestral population. Colonization northwards from NCA to NChiapas: NCA > TM > SMO > NChiapas. At t2 admixture between SMO and NChiapas populations originates SMS population.
15. Divergence of SCA and an ancestral population at t6. Northward population expansion from NCA to Mexico. At t2 admixture between SMO and NChiapas populations originates SMS population.


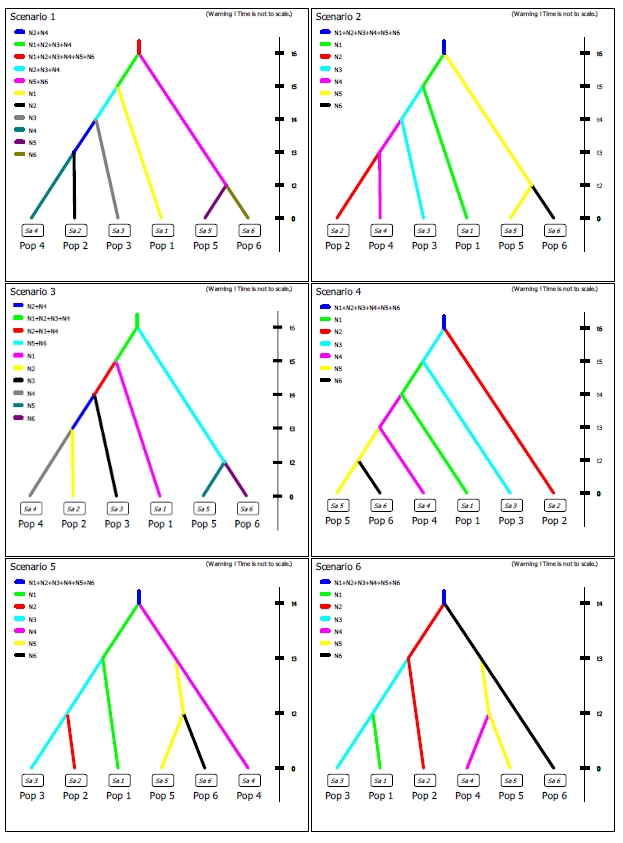


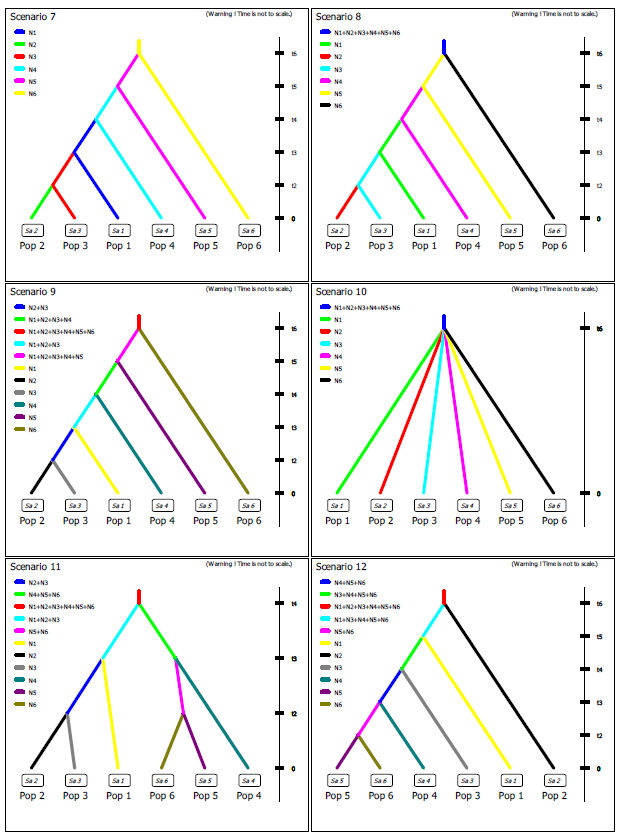


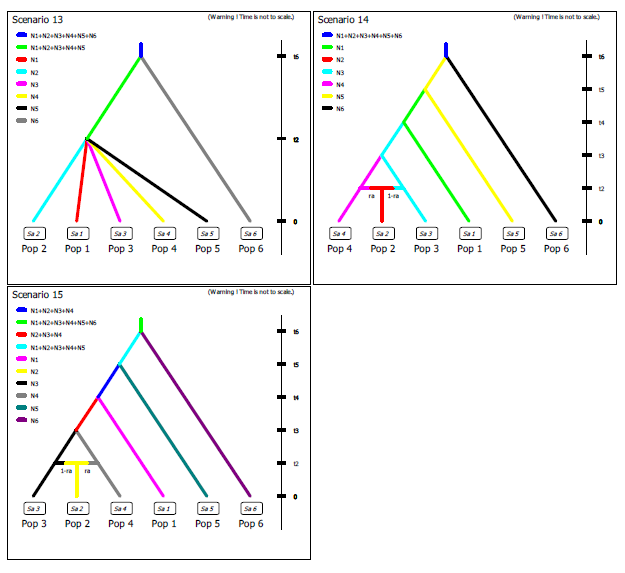


Figure 2. Scenarios tested with ABC for *Chlorospingus*. Numbers for each scenario correspond to the hypothesis in tested scenarios.

***Cardellina***

Populations:

1: *C. versicolor* (northern Central America)

2: SMS (Oaxaca)

3: SMOc (Sierra Madre Occidental)

4: TMVB (Trans Mexican Volcanic Belt)

Tested scenarios: *H0 scenario, **selected real scenario.

1. **Divergence between *C. versicolor* and SMOc populations at t4, then divergence between SMOc and an hypothetical ancestral population from SMS+TMVB. At t2 divergence between SMS and TMVB populations followed by an effective population change in TMVB at t1.
2. Divergence between *C. versicolor* and a hypothetical ancestral population. At t3 divergence between SMOc and an ancestral population SMS+TMVB. At t2 divergence between SMS and TMVB populations, while both at t1 have an effective population size change.
3. Divergence between *C. versicolor* and TMVB at t4. Simultaneous divergence of SMOc and SMS at t2. Effective population size change at t1 for TMVB population.
4. Divergence at t4 of *C. versicolor* and TMVB. Simultaneous divergence of SMOc and SMS at t2. Effective population size change at t1 for TMVB and SMS populations.
5. *Divergence of *C. versicolor* and an hypothetical ancestral population of SMS+TMVB+SMOc. Simultaneous diversification at t2 of SMS, SMOc and TMVB populations.
6. Divergence between *C. versicolor* and an *C. rubra* ancestral population at t4. Divergence of SMOc and an ancestral population of SMS+TMVB at t3. At t2 divergence between SMS and TMVB. Population size change of TMVB at t1.
7. Divergence between *C. versicolor* and TMVB at t4. Divergence between TMVB and an ancetral population of SMS+TMVB at t3. Divergence between SMS and TMVB at t2 with population expansion of TMVB at t1.
8. Divergence between *C. versicolor* and TMVB populations at t4. At t3 populations from SMOc are originated from TMVB population at t3. At t2 occurs admixture between SMOc and TMVB that originates SMS population.
9. Divergence of *C. versicolor* and a hypothetical ancestral population, from which SMOc and TMVB populations diverge at t3. TMVB and SMOc have genetic admixture which originates SMS population at t2.
10. Divergence at t4 of *C. versicolor* and an ancestral population from TMVB+SMS+SMOc. Divergence between TMVB and SMOc populations at t3, then SMS population originates from TMVB population at t2. Effective population size change of TMVB population at t1.
11. Divergence between *C. versicolor* and an hypothetical ancestral population. At t3 divergence between SMOc and an ancestral population of SMS+TMVB. At t2 divergence between SMS and TMVB, and then TMVB has a population size change.
12. Divergence of *C. versicolor* and TMVB from an hypothetical ancestral population at t4. TMVB population at t3 colonizes SMOc and at t2 colonizes SMS. Population size change at t1 of TMVB population.
13. Divergence of *C. versicolor* and a hypothetical ancestral population at t4. At t3 divergence between SMS and TMVB populations. TMVB colonizes at t3 SMOc and then has an effective population size change.
14. Divergence between *C. versicolor* and TMVB from an hypothetical ancestral population at t4. TMVB population at t3 colonizes SMS and at t2 colonizes SMOc. Population size change at t1 of TMVB population.
15. Divergence of *C. versicolor* and SMS populations from an ancestral population at t4. Colonization northwards from SMS to SMOc: SMS > TMVB > SMOc. At t1 population size change of TMVB population.


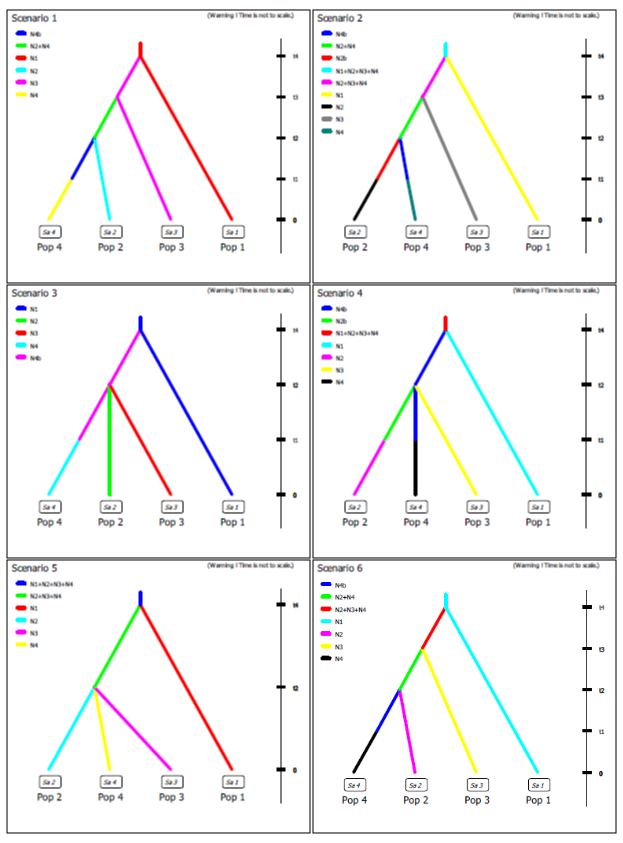


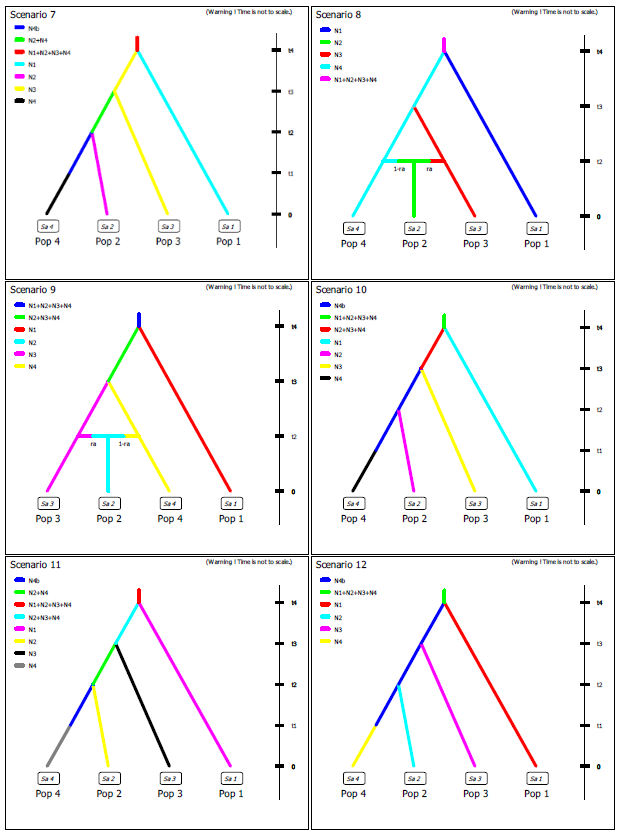


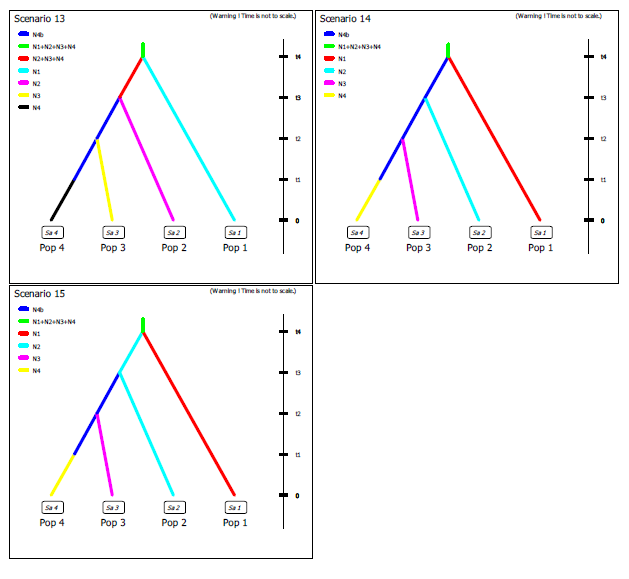


Figure 3. Scenarios tested with ABC for *Cardellina*. Numbers for each scenario correspond to the hypothesis in tested scenarios.

***Eupherusa***

Populations:

1: *E. nigriventris*(Costa Rica-Panama)

2: *E. eximia* (northern Oaxaca-north Central America)

3: *E. cyanoprhys* (Sierra de Miahuatlán, Oaxaca)

4: *E. poliocerca* (Guerrero-Oaxaca)

5: *Thalurania ridgwayi* (Jalisco)

Tested scenarios: *H0 scenario, **selected real scenario.

1. Divergence of *T. ridgwayi* and an hypothetical ancestral population of *Eupherusa* at t4. Ancestral *Eupherusa* diverges at t3 into an ancestral population of *E. nigriventris*+*E. eximia*, and an ancestral population of *E. poliocerca*+*E. cyanoprhys*; which diverge at t2 and t1 respectively.
2. **Divergence between *T. ridgwayi* and an hypothetical ancestral population of *Eupherusa* at t4. Ancestral *Eupherusa* diverges at t3 into an ancestral population of E. nigriventris+*E. eximia*, and an ancestral population of *E. poliocerca*+*E. cyanoprhys*; which diverge at t2 and t1 respectively.
3. Divergence between *T. ridgwayi* and *Eupherusa poliocerca* at t4. Then population of *E. eximia* was originated fromo *E. poliocerca* at t3. *E. nigriventris* was originated from *E. eximia* population at t2, and then at t1 *E. cyanoprhys* is orginated from *E. policerca*.
4. Divergence of *T. ridgwayi* and an hypothetical ancestral population of *Eupherusa*. At t3 *E*. *eximia* and *E. poliocerca* diverge. At t2 *E. nigriventris* population was originated from *E*. *eximia*, and then at t1 *E. cyanorphrys* was originated from *E. poliocerca* population.
5. Divergence between *T. ridgwayi* and an hypothetical ancestral *Eupherusa* population at t4. At t3 ancestral *Eupherusa* diverge into ancestral *E. cyanoprhys*+*E. policerca* and *E. nigriventris*+*E. eximia*; which then at t1 diverge.
6. Divergence between *T. ridgwayi* and *E. nigriventris* at t4. At t3 there’s a northward colonization which resulted in the divergence of *E. policoerca* at t3, and *E. eximia* at t2. At t1 *E. cyanoprhys* is originated from *E. policerca* population.
7. Divergence of *T. ridgwayi* and *E. nigriventris* from an ancestral population at t4. Northward colonization resulted in divergence at t3 of E. poliocerca and *E. eximia* at t2 from *E. nigriventris* population. At t1 *E. cyanorphys* is originated from *E. poliocerca*.
8. Divergence of *E. cyanoprhys* and *E. eximia* from an ancestral population at t3. E. poliocerca diverges at t2 from *E. cyanoprhys* and *T. ridgwayi* is originated from *E. poliocerca* at t1; while *E. nigriventris* originates from *E. eximia*.
9. Divergence between *T. ridgwayi* and *E. eximia* from an ancestral population at t3. *E. poliocerca* originates from *T. ridgwayi* at t2 and then *E. cyanprhys* orignates from *E. poliocerca* at t1; while *E. nigriventris* originates from *E. eximia*.
10. Divergence between *E. nigriventris* and *E. eximia* at t4. Succesive founder events *E. eximia* > *E. cyanoprhys* > *E. poliocerca* > *T. ridgwayi*.
11. Divergence between *E. nigriventris* and a hypothetical ancestral population of *E. eximia* + *E. cyanoprhys* + *E. poliocerca* + *T. ridgwayi.* Divergence of *E. eximia* at t3 and an ancestral population. At t2 *E. cyanoprhys* and an hypothetical ancestral population of *E. poliocerca* + *T. ridgwayi* diverge, then divergence between *E. poliocerca* and *T. ridgwayi*.
12. Divergence between *T. ridgwayi* and *E. poliocerca* at t4. Succesive founder events *E. poliocerca* > *E. cyanoprhys* > *E. eximia* > *E. nigriventris.*
13. Divergece at t4 of *T. ridgwayi* and an ancestral population of *Eupherusa*. Divergence of *E. poliocerca* and an ancestral population of *E. eximia*+*E. nigriventris*+*E. cyanoprhys* at t3. Divergence of *E. cyanoprhys* and an ancestral population of *E. eximia*+*E. nigriventris* at t2., then divergence between *E. eximia* and *E. nigriventris*.
14. *Simultaneous divergence from an ancestral population at t4 from *Eupherusa* and *Thalurania ridgwayi* populations.
15. Divergence of *T. ridgwayi* and a hypothetical ancestral *Eupherusa* population at t4. Simultaneous divergence of *Eupherusa* species at t3.


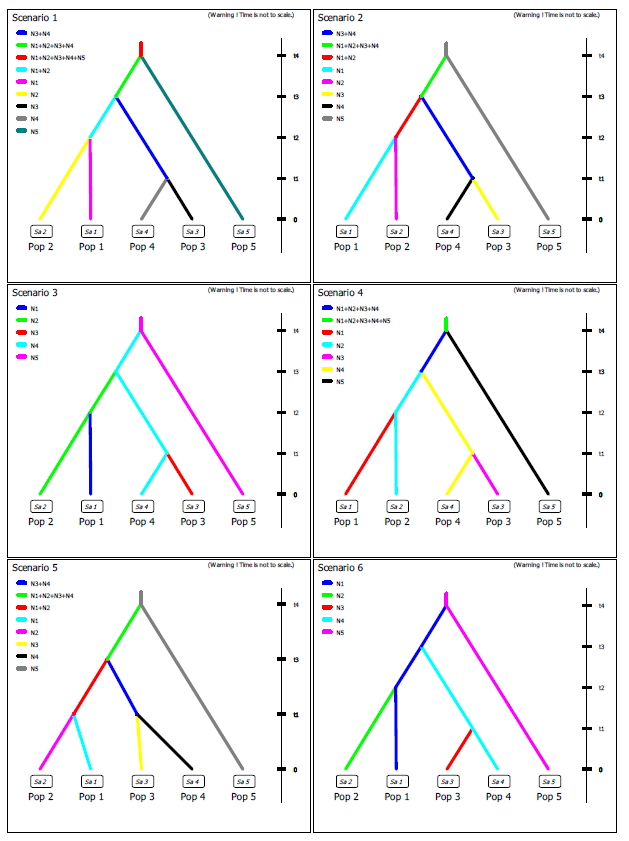


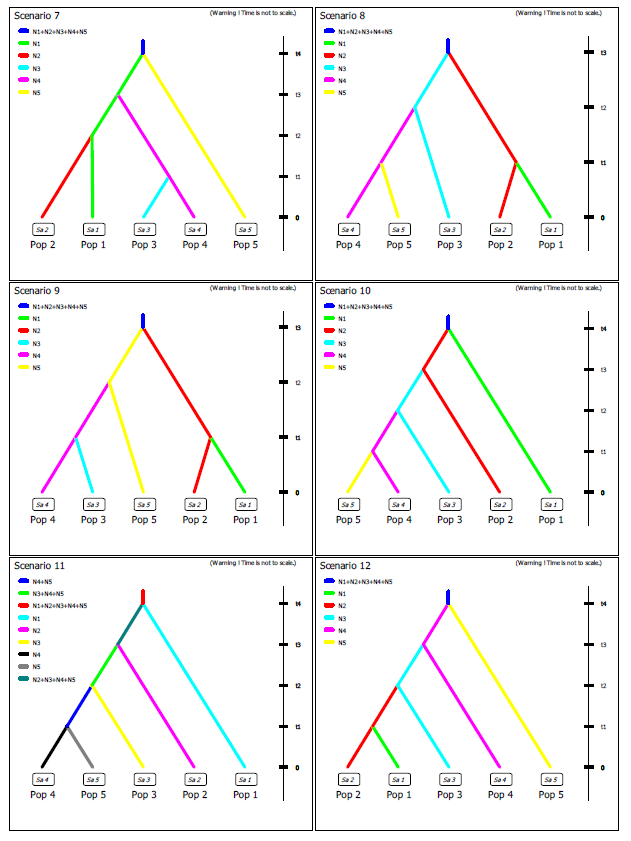


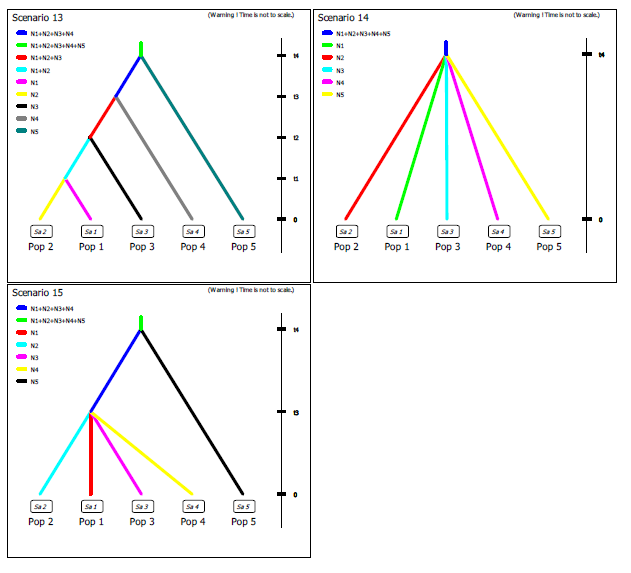


Figure 4. Scenarios tested with ABC for *Eupherusa*. Numbers for each scenario correspond to the hypothesis in tested scenarios.
