# Supplementary material for: The geography of evolutionary divergence in the highly endemic avifauna from the Sierra Madre del Sur, Mexico
Source: BMC Evol Biol. 2019 Dec 30;19:237. doi: 10.1186/s12862-019-1564-3 (PMC6937948; doi:10.1186/s12862-019-1564-3)
Supplement: Supplementary file 4 — Additional file 4: Posterior parameter estimates for the best supported scenarios. Population and divergence time estimates obtained with ABC for Aulacorhynchus, Chlorospingus, Cardellina, and Eupherusa-Thalurania. (DOCX 33 kb) [file 12862_2019_1564_MOESM4_ESM.docx]

**The geography of evolutionary divergence in the highly endemic avifauna from the Sierra Madre del Sur, Mexico**

ALBERTO ROCHA-MÉNDEZ, LUIS A. SÁNCHEZ-GONZÁLEZ, CLEMENTINA GONZÁLEZ, & ADOLFO G. NAVARRO-SIGÜENZA

**Supporting Information**

Table 5. Posterior parameter estimates for the best supported scenario (scenario 1) considering four groups in *Aulacorhynchus*. Estimates are based on the 1% of simulated datasets closest to the observed values. Parameters delineate as follows: N=effective population size for EMx-NCA (N_1_), SMS (N_2_), CR-NP (N_3_), SP (N_4_), *t*=time since divergence (generations).

| Parameter | Mean | Median | Mode | 2.5% | 97.5% |
| --- | --- | --- | --- | --- | --- |
| N1 | 4.72 × 10^5^ | 4.52 × 10^5^ | 3.99 × 10^5^ | 1.87 × 10^5^ | 8.44 × 10^5^ |
| N2 | 6.91 × 10^5^ | 6.74 × 10^5^ | 6.67 × 10^5^ | 3.05 × 10^5^ | 1.17 × 10^6^ |
| N3 | 4.54 × 10^4^ | 4.57 × 10^4^ | 4.47 × 10^4^ | 1.53 × 10^4^ | 7.23 × 10^4^ |
| N4 | 2.83 × 10^4^ | 2.8 × 10^4^ | 2.53 × 10^4^ | 1.81× 10^3^ | 5.72 × 10^4^ |
| t2 | 6.64 × 10^5^ | 6.46 × 10^5^ | 6.74 × 10^5^ | 2.1 × 10^5^ | 1.25 × 10^6^ |
| t3 | 1.43 × 10^6^ | 1.42 × 10^6^ | 1.41× 10^6^ | 1.23 × 10^6^ | 1.71 × 10^6^ |
| t4 | 2.17 × 10^6^ | 2.15 × 10^6^ | 2.11 × 10^6^ | 2.04 × 10^6^ | 2.4 × 10^6^ |

Table 6. Posterior parameter estimates for the best supported scenario (scenario 2) considering five groups in *Aulacorhynchus*. Estimates are based on the 1% of simulated datasets closest to the observed values. Parameters delineate as follows: N=effective population size for EMx-NCA (N_1_), SMS-Guerrero (N_2_), SMS-Oaxaca (N_3_), CR-NP (N_4_), SP (N_5_), *t*=time since divergence (generations).

| Parameter | Mean | Median | Mode | 2.5% | 97.5% |
| --- | --- | --- | --- | --- | --- |
| N1 | 4.57 × 10^5^ | 4.17 × 10^5^ | 3.63 × 10^5^ | 1.73 × 10^5^ | 9.7 × 10^5^ |
| N2 | 6.83 × 10^4^ | 3.78 × 10^4^ | 6.57 × 10^3^ | 1.91 × 10^3^ | 3.06 × 10^5^ |
| N3 | 7.78 × 10^5^ | 7.59 × 10^5^ | 7 × 10^5^ | 3.71 × 10^5^ | 1.28 × 10^6^ |
| N4 | 3.65 × 10^4^ | 3.35 × 10^4^ | 2.56 × 10^4^ | 3.68 × 10^3^ | 8.13 × 10^4^ |
| N5 | 3.52 × 10^4^ | 3.53 × 10^4^ | 3.45 × 10^4^ | 4.36 × 10^3^ | 6.57 × 10^4^ |
| t1 | 1.02 × 10^5^ | 7.34 × 10^4^ | 2.12 × 10^4^ | 6.4 × 10^3^ | 3.66 × 10^5^ |
| t2 | 9.42 × 10^5^ | 8.67 × 10^5^ | 8.69 × 10^5^ | 2.68 × 10^5^ | 2.2 × 10^6^ |
| t3 | 1.15 × 10^6^ | 1.05 × 10^6^ | 9.79 × 10^5^ | 6.83 × 10^5^ | 2.44 × 10^6^ |
| t4 | 1.65 × 10^6^ | 1.5 × 10^6^ | 1.43 × 10^6^ | 1.29 × 10^6^ | 3.09 × 10^6^ |

Table 7. Posterior parameter estimates for the best supported scenario (scenario 1) considering six groups in *Chlorospingus*. Estimates are based on the 1% of simulated datasets closest to the observed values. Parameters delineate as follows: N=effective population size for Tuxtlas Massif (N_1_), SMS (N_2_), SMO (N_3_), N Chiapas (N_4_), NCA (N_5_), SCA (N_6_), *t*=time since divergence (generations).

| Parameter | Mean | Median | Mode | 2.5% | 97.5% |
| --- | --- | --- | --- | --- | --- |
| N1 | 2.34 × 10^6^ | 2.2 × 10^6^ | 1.65 × 10^6^ | 7.1 × 10^5^ | 4.61 × 10^6^ |
| N2 | 1.78 × 10^6^ | 1.6 × 10^6^ | 1.3 × 10^6^ | 4.49 × 10^5^ | 3.99 × 10^6^ |
| N3 | 2.17 × 10^6^ | 2.19 × 10^6^ | 2.04 × 10^6^ | 1.34 × 10^6^ | 2.88 × 10^6^ |
| N4 | 1.18 × 10^6^ | 1.1 × 10^6^ | 8.4 × 10^5^ | 2.56 × 10^5^ | 2.49 × 10^6^ |
| N5 | 1.68 × 10^6^ | 1.61 × 10^6^ | 1.53 × 10^6^ | 6.22 × 10^5^ | 3.13 × 10^6^ |
| N6 | 7.3 × 10^5^ | 6.45 × 10^5^ | 3.84 × 10^5^ | 1.28 × 10^5^ | 1.75 × 10^6^ |
| t2 | 6.54 × 10^5^ | 6.31 × 10^5^ | 5.9 × 10^5^ | 6.94 × 10^4^ | 1.37 × 10^6^ |
| t3 | 1.03 × 10^6^ | 1.03 × 10^6^ | 1.07 × 10^6^ | 3.86 × 10^5^ | 1.73 × 10^6^ |
| t4 | 1.42 × 10^6^ | 1.41 × 10^6^ | 1.37 × 10^6^ | 6.71 × 10^5^ | 2.27 × 10^6^ |
| t5 | 1.95 × 10^6^ | 1.89 × 10^6^ | 1.57 × 10^6^ | 8.99 × 10^5^ | 3.26 × 10^6^ |
| t6 | 2.93 × 10^6^ | 2.85 × 10^6^ | 2.52 × 10^6^ | 1.26 × 10^6^ | 4.78 × 10^6^ |

Table 8. Posterior parameter estimates for the best supported scenario (scenario 1) considering seven groups in *Chlorospingus*. Estimates are based on the 1% of simulated datasets closest to the observed values. Parameters delineate as follows: N=effective population size for Tuxtlas Massif (N_1_), SMS-Guerrero (N_2_), SMS-Oaxaca (N_3_), SMO (N_4_), N Chiapas (N_5_), NCA (N_6_), SCA (N_7_), *t*=time since divergence (generations).

| Parameter | Mean | Median | Mode | 2.5% | 97.5% |
| --- | --- | --- | --- | --- | --- |
| N1 | 3.6 × 10^6^ | 3.67 × 10^6^ | 3.82 × 10^6^ | 1.95 × 10^6^ | 4.86 × 10^6^ |
| N2 | 1.57 × 10^6^ | 1.59 × 10^6^ | 1.64 × 10^6^ | 6.11 × 10^5^ | 2.39 × 10^6^ |
| N3 | 2.14 × 10^5^ | 1.96 × 10^5^ | 5.72 × 10^4^ | 1.14 × 10^4^ | 4.78 × 10^5^ |
| N4 | 2.73 × 10^6^ | 2.76 × 10^6^ | 2.84 × 10^6^ | 1.98 × 10^6^ | 3.29 × 10^6^ |
| N5 | 7.99 × 10^5^ | 6.68 × 10^5^ | 4.28 × 10^5^ | 1.45 × 10^5^ | 2.2 × 10^6^ |
| N6 | 2.61 × 10^6^ | 2.66 × 10^6^ | 2.88 × 10^6^ | 1.45 × 10^6^ | 3.44 × 10^6^ |
| N7 | 6.82 × 10^5^ | 5.58 × 10^5^ | 3.73 × 10^5^ | 1.1× 10^5^ | 1.95 × 10^6^ |
| t1 | 3.28 × 10^5^ | 2.91 × 10^5^ | 1.72 × 10^5^ | 2.5 × 10^4^ | 8.23 × 10^5^ |
| t2 | 5.07 × 10^5^ | 4.81 × 10^5^ | 4.47 × 10^5^ | 1.44 × 10^5^ | 1.02 × 10^6^ |
| t3 | 6.09 × 10^5^ | 5.82 × 10^5^ | 5.09 × 10^5^ | 2.58 × 10^5^ | 1.13 × 10^6^ |
| t4 | 7.99 × 10^5^ | 7.28 × 10^5^ | 6.65 × 10^5^ | 3.08 × 10^5^ | 1.7 × 10^6^ |
| t5 | 9.73 × 10^5^ | 8.69 × 10^5^ | 7.45 × 10^5^ | 5.99 × 10^5^ | 2.1 × 10^6^ |
| t6 | 1.02 × 10^6^ | 8.65 × 10^5^ | 7.35 × 10^5^ | 5.92 × 10^5^ | 2.67 × 10^6^ |

Table 9. Posterior parameter estimates for the best supported scenario (scenario 3) considering four groups in *Cardellina*. Estimates are based on the 1% of simulated datasets closest to the observed values. Parameters delineate as follows: N=effective population size for *C. versicolor* (N_1_), SMS (N_2_), SMOc (N_3_), TMVB (N_4_), *t*=time since divergence (generations).

| Parameter | Mean | Median | Mode | 2.5% | 97.5% |
| --- | --- | --- | --- | --- | --- |
| N1 | 2.21 × 10^5^ | 1.65 × 10^5^ | 9.11 × 10^4^ | 3.51 × 10^4^ | 6.74 × 10^5^ |
| N2 | 2.13 × 10^5^ | 1.98 × 10^5^ | 1.67 × 10^5^ | 9.17 × 10^4^ | 4.18 × 10^5^ |
| N3 | 4.76 × 10^5^ | 3.71 × 10^5^ | 2.09 × 10^5^ | 6.75 × 10^4^ | 1.46 × 10^6^ |
| N4 | 2.61 × 10^5^ | 2.55 × 10^5^ | 2.47 × 10^5^ | 1.37 × 10^5^ | 4.23 × 10^5^ |
| N4b | 6.96 × 10^4^ | 6.36 × 10^4^ | 4.96 × 10^4^ | 5.46 × 10^3^ | 1.73 × 10^5^ |
| t1 | 2.01 × 10^4^ | 1.65 × 10^4^ | 1.04 × 10^4^ | 1.75 × 10^3^ | 6.1 × 10^4^ |
| t2 | 1.3 × 10^5^ | 1.27 × 10^5^ | 1.17 × 10^5^ | 5.69 × 10^4^ | 2.16 × 10^5^ |
| t3 | 2.13 × 10^5^ | 2.11 × 10^5^ | 1.94 × 10^5^ | 1.12 × 10^5^ | 3.31 × 10^5^ |
| t4 | 6.52 × 10^5^ | 4.89 × 10^5^ | 3.89 × 10^5^ | 2.06 × 10^5^ | 2.18 × 10^6^ |

Table 10. Posterior parameter estimates for the best supported scenario (scenario 2) considering five groups in *Eupherusa* including *T. ridgwayi*. Estimates are based on the 1% of simulated datasets closest to the observed values. Parameters delineate as follows: N=effective population size for *nigriventris* (N_1_), *eximia* (N_2_), *cyanophrys* (N_3_), *poliocerca* (N_4_), *T. ridgwayi* (N_5_), *t*=time since divergence (generations).

| Parameter | Mean | Median | Mode | 2.5% | 97.5% |
| --- | --- | --- | --- | --- | --- |
| N1 | 2.33 × 10^5^ | 1.9 × 10^5^ | 7.44 × 10^4^ | 1.31 × 10^4^ | 6.34 × 10^5^ |
| N2 | 1.12 × 10^5^ | 9.04 × 10^4^ | 4.77 × 10^4^ | 1.03 × 10^4^ | 3.47 × 10^5^ |
| N3 | 6.06 × 10^5^ | 5.79 × 10^5^ | 5.57 × 10^5^ | 2.53 × 10^5^ | 1.12 × 10^6^ |
| N4 | 1.87 × 10^5^ | 1.66 × 10^5^ | 1.05 × 10^5^ | 2.34 × 10^4^ | 4.69 × 10^5^ |
| N5 | 2.03 × 10^5^ | 1.55 × 10^5^ | 9.39 × 10^4^ | 1.31 × 10^4^ | 6.7 × 10^5^ |
| t1 | 3.58 × 10^5^ | 3.36 × 10^5^ | 2.88 × 10^5^ | 6.17 × 10^4^ | 7.6 × 10^5^ |
| t2 | 6.73 × 10^5^ | 6.78 × 10^5^ | 7.28 × 10^5^ | 3.17 × 10^5^ | 1 × 10^6^ |
| t3 | 9.02 × 10^5^ | 9.02 × 10^5^ | 8.91 × 10^5^ | 6.47 × 10^5^ | 1.16 × 10^6^ |
| t4 | 1.39 × 10^6^ | 1.39 × 10^6^ | 1.35 × 10^6^ | 1.07 × 10^6^ | 1.71 × 10^6^ |

Table 11. Posterior parameter estimates for the best supported scenario (scenario 1) considering four groups in *Eupherusa* matrix excluding *T. ridgwayi*. Estimates are based on the 1% of simulated datasets closest to the observed values. Parameters delineate as follows: N=effective population size for *nigriventris* (N_1_), *eximia* (N_2_), *cyanophrys* (N_3_), *poliocerca* (N_4_), *t*=time since divergence (generations).

| Parameter | Mean | Median | Mode | 2.5% | 97.5% |
| --- | --- | --- | --- | --- | --- |
| N1 | 2.7 × 10^5^ | 2.4 × 10^5^ | 1.07 × 10^5^ | 1.16 × 10^4^ | 6.56 × 10^5^ |
| N2 | 1.3 × 10^5^ | 1.01 × 10^5^ | 6.07 × 10^4^ | 9.49 × 10^3^ | 4.11 × 10^5^ |
| N3 | 6.25 × 10^5^ | 5.98 × 10^5^ | 5.44 × 10^5^ | 2.61 × 10^5^ | 1.13 × 10^6^ |
| N4 | 1.65 × 10^5^ | 1.41 × 10^5^ | 8.88 × 10^4^ | 1.62 × 10^4^ | 4.43 × 10^5^ |
| t1 | 3.44 × 10^5^ | 3.22 × 10^5^ | 2.72 × 10^5^ | 5.8 × 10^4^ | 7.54 × 10^5^ |
| t2 | 6.45 × 10^5^ | 6.44 × 10^5^ | 6.21 × 10^5^ | 2.97 × 10^5^ | 1.01 × 10^6^ |
| t3 | 8.62 × 10^5^ | 8.63 × 10^5^ | 8.78 × 10^5^ | 5.39 × 10^5^ | 1.19 × 10^6^ |
