# Supplementary material for: The geography of evolutionary divergence in the highly endemic avifauna from the Sierra Madre del Sur, Mexico
Source: BMC Evol Biol. 2019 Dec 30;19:237. doi: 10.1186/s12862-019-1564-3 (PMC6937948; doi:10.1186/s12862-019-1564-3)
Supplement: Supplementary file 5 — Additional file 5: Final tested hypothetical scenarios of diversification of 4 bird taxa in Mesoamerica. Competing demographic scenarios of Aulacorhynchus, Chlorospingus, Cardellina, and Eupherusa-Thalurania with posterior probabilities and model checking. [file 12862_2019_1564_MOESM5_ESM.docx]

**The geography of evolutionary divergence in the highly endemic avifauna from the Sierra Madre del Sur, Mexico**

ALBERTO ROCHA-MÉNDEZ, LUIS A. SÁNCHEZ-GONZÁLEZ, CLEMENTINA GONZÁLEZ, & ADOLFO G. NAVARRO-SIGÜENZA

**Supporting Information**

*Final tested hypothetical scenarios of diversification of 4 bird taxa in Mesoamerica.*


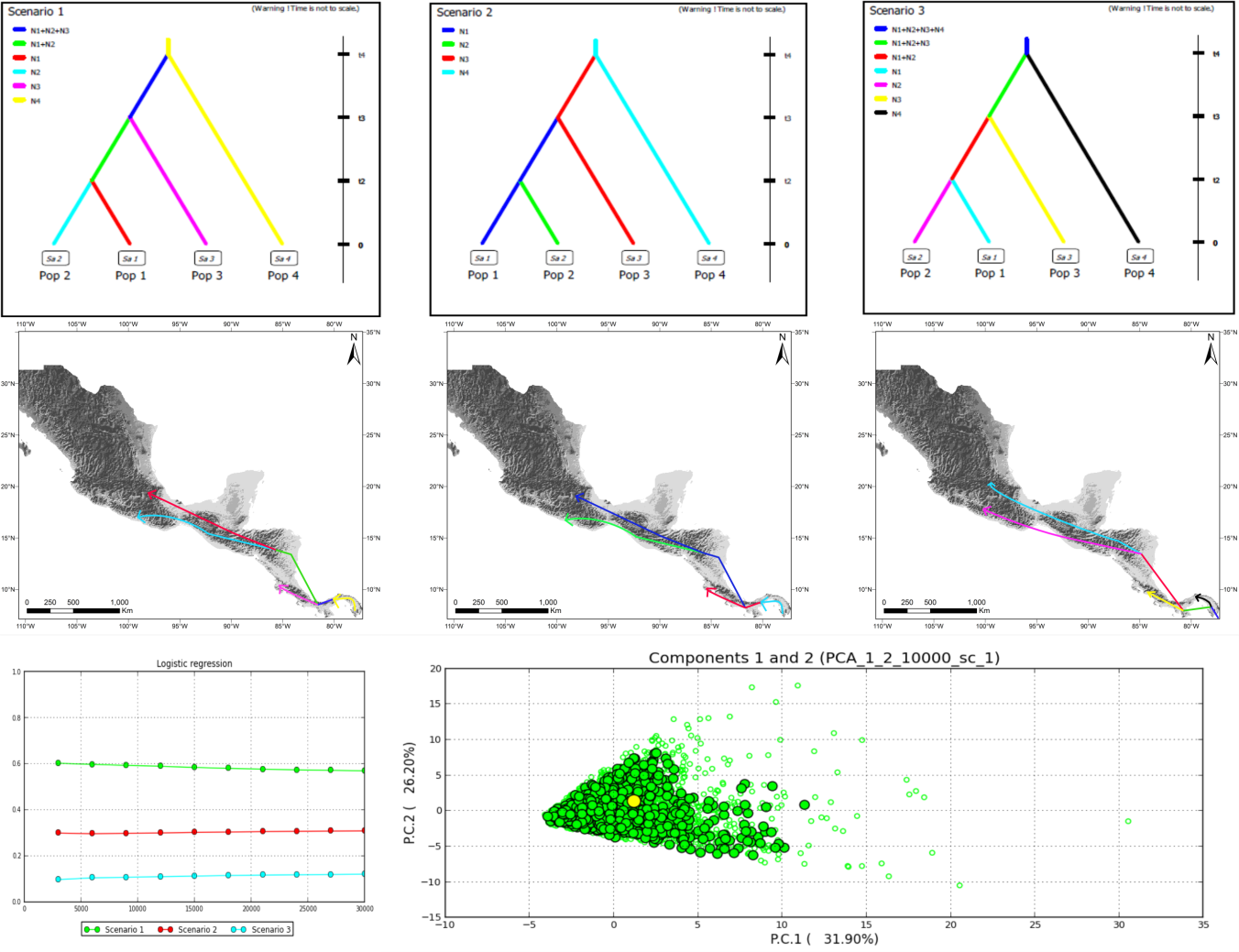


Fig. 5: Competing demographic scenarios of *Aulacorhynchus* with four populations, posterior probability of scenarios assessed with a logistic regression on the 1% of the simulated datasets closest to the observed data, and model checking for the best supported scenario (scenario 1) applying a PCA on test statistic vectors to visualize fit between simulated and observed datasets.


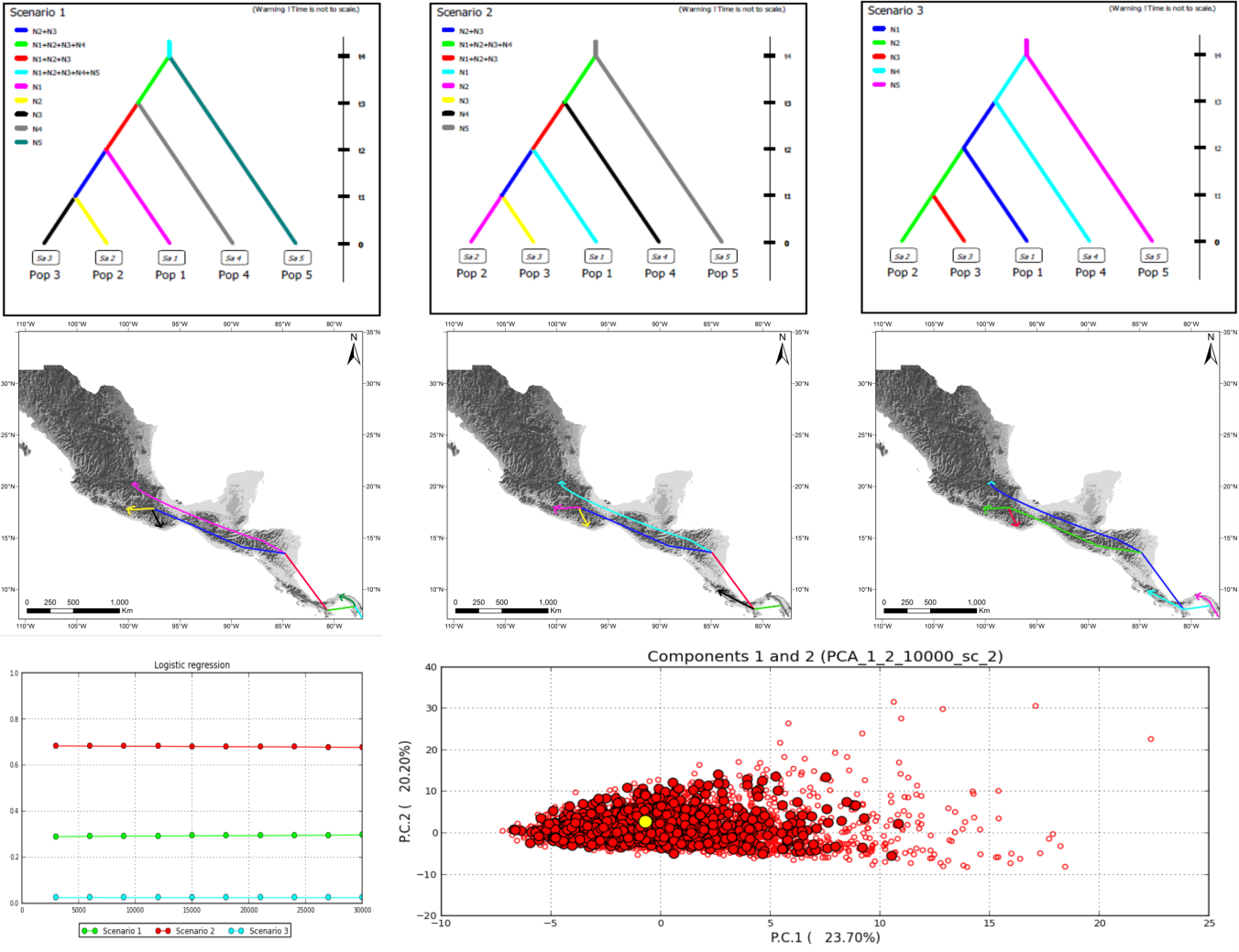


Fig. 6: Competing demographic scenarios of *Aulacorhynchus* with five populations, posterior probability of scenarios assessed with a logistic regression on the 1% of the simulated datasets closest to the observed data, and model checking for the best supported scenario (scenario 2) applying a PCA on test statistic vectors to visualize fit between simulated and observed datasets.


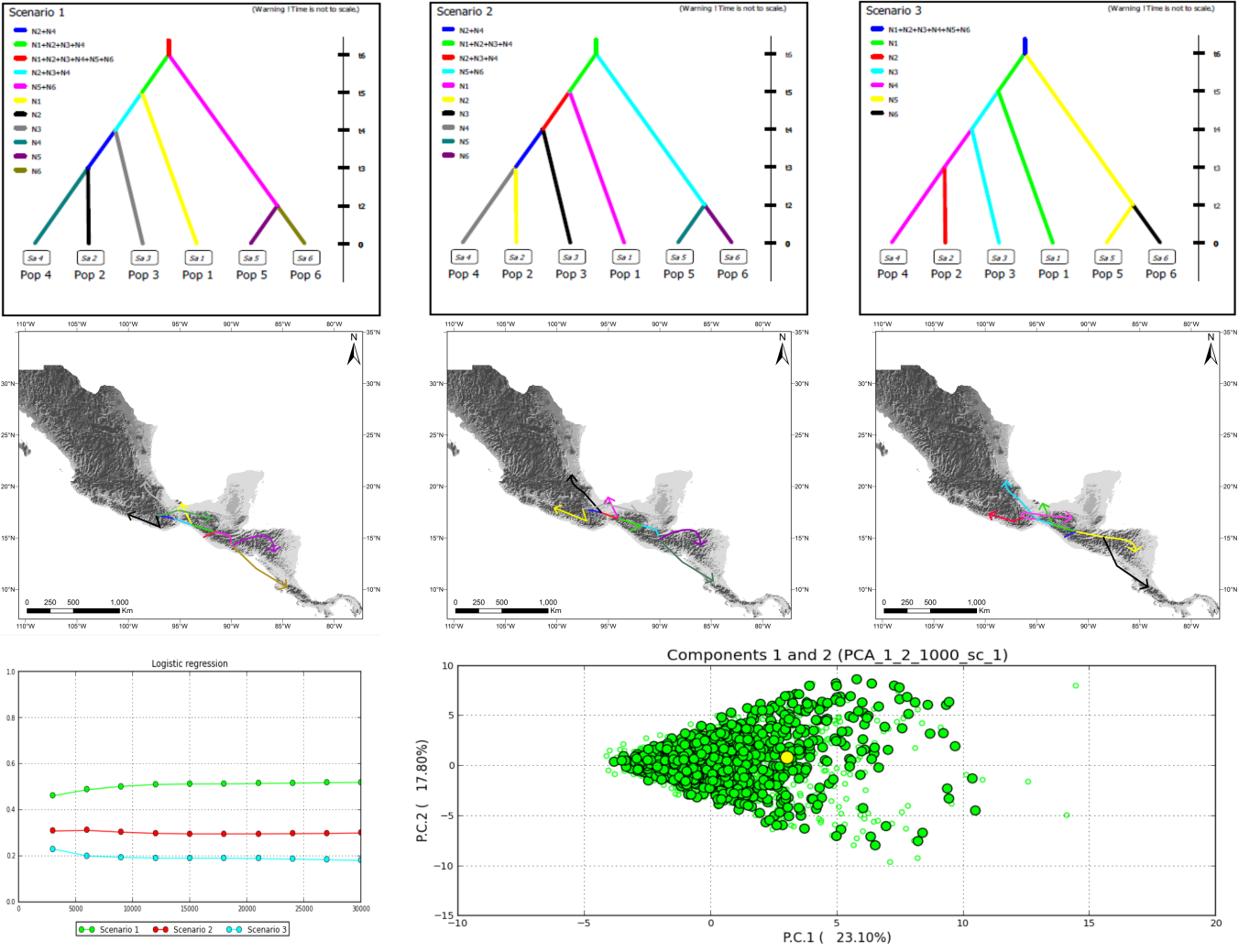


Fig. 7: Competing demographic scenarios of *Chlorospingus* with six populations, posterior probability of scenarios assessed with a logistic regression on the 1% of the simulated datasets closest to the observed data, and model checking for the best supported scenario (scenario 1) applying a PCA on test statistic vectors to visualize fit between simulated and observed datasets.


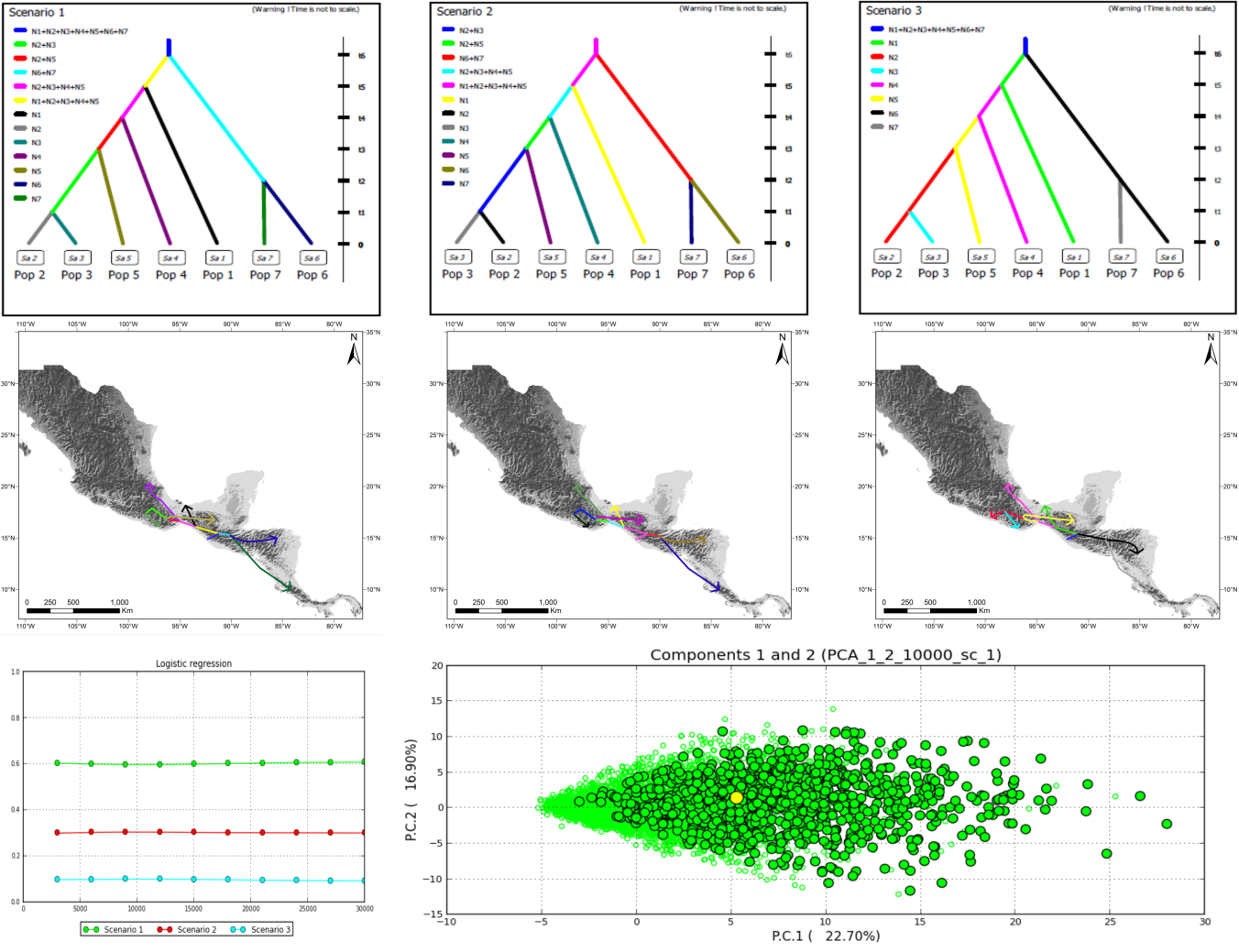


Fig. 8: Competing demographic scenarios of *Chlorospingus* with seven populations, posterior probability of scenarios assessed with a logistic regression on the 1% of the simulated datasets closest to the observed data, and model checking for the best supported scenario (scenario 1) applying a PCA on test statistic vectors to visualize fit between simulated and observed datasets.


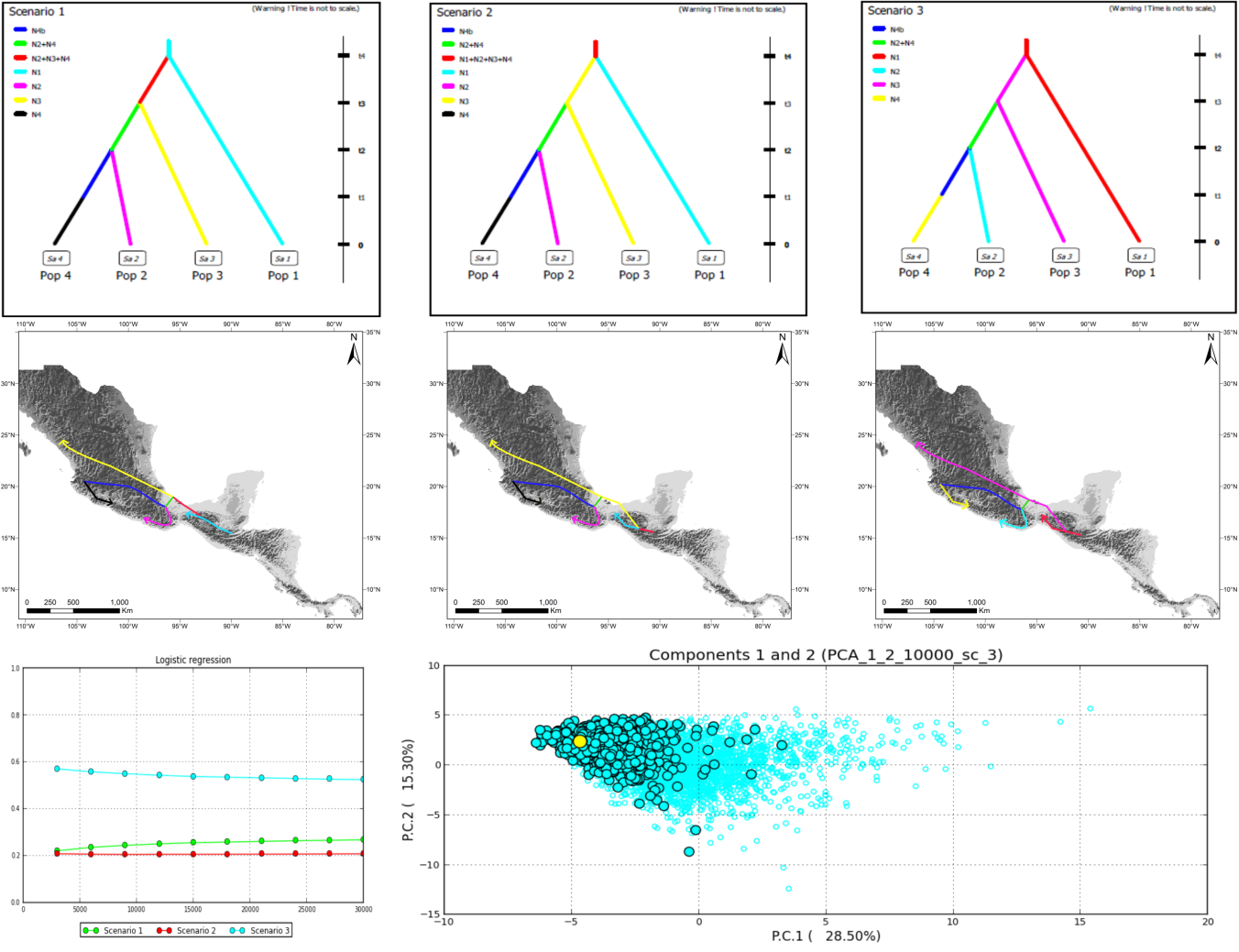


Fig. 9: Competing demographic scenarios of *Cardellina*, posterior probability of scenarios assessed with a logistic regression on the 1% of the simulated datasets closest to the observed data, and model checking for the best supported scenario (scenario 3) applying a PCA on test statistic vectors to visualize fit between simulated and observed datasets.


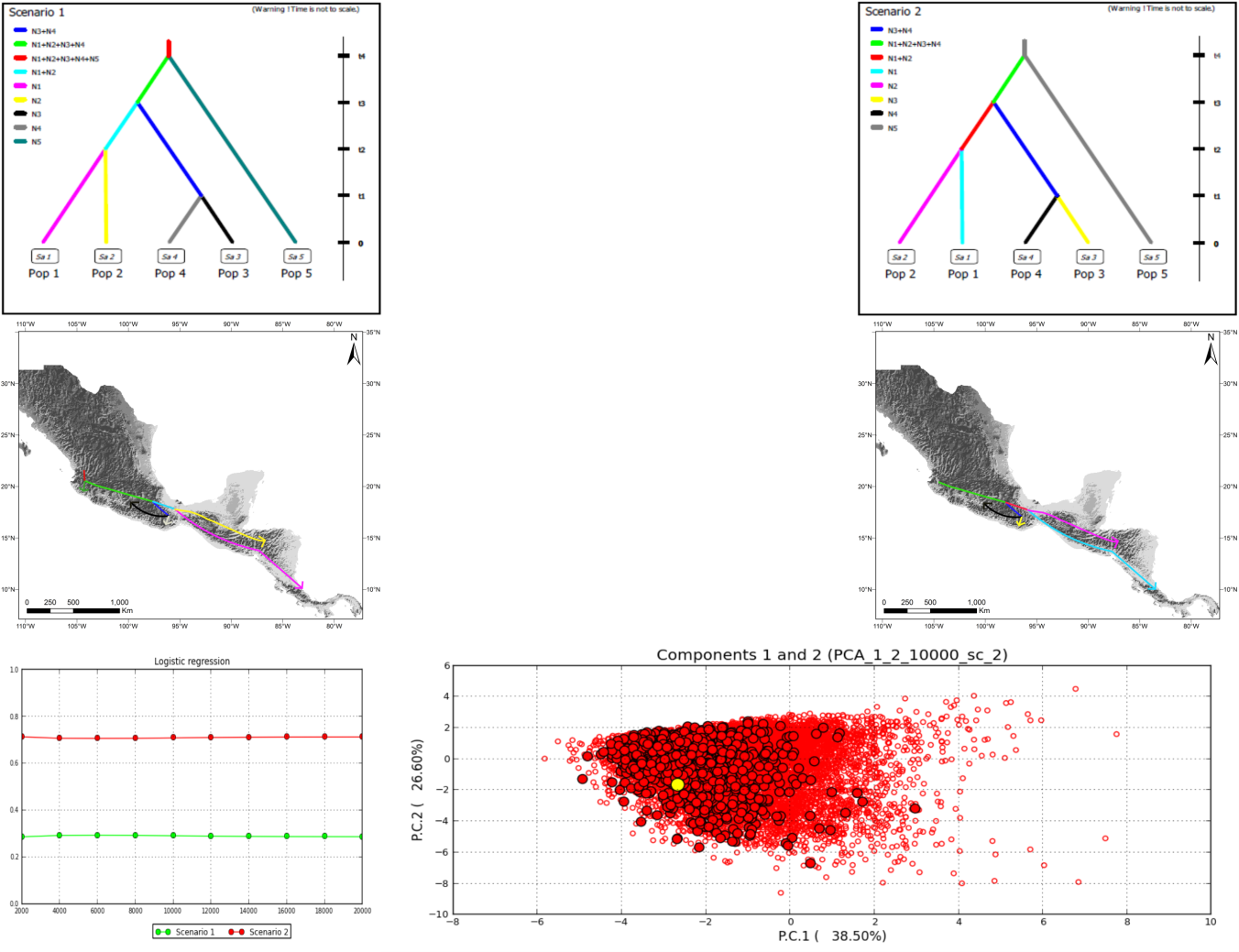


Fig. 10. Competing scenarios of *Eupherusa-Thalurania*, posterior probability of scenarios assessed with a logistic regression on the 1% of the simulated datasets closest to the observed data, and model checking for the best supported scenario (scenario 2) applying a PCA on test statistic vectors to visualize fit between simulated and observed datasets.


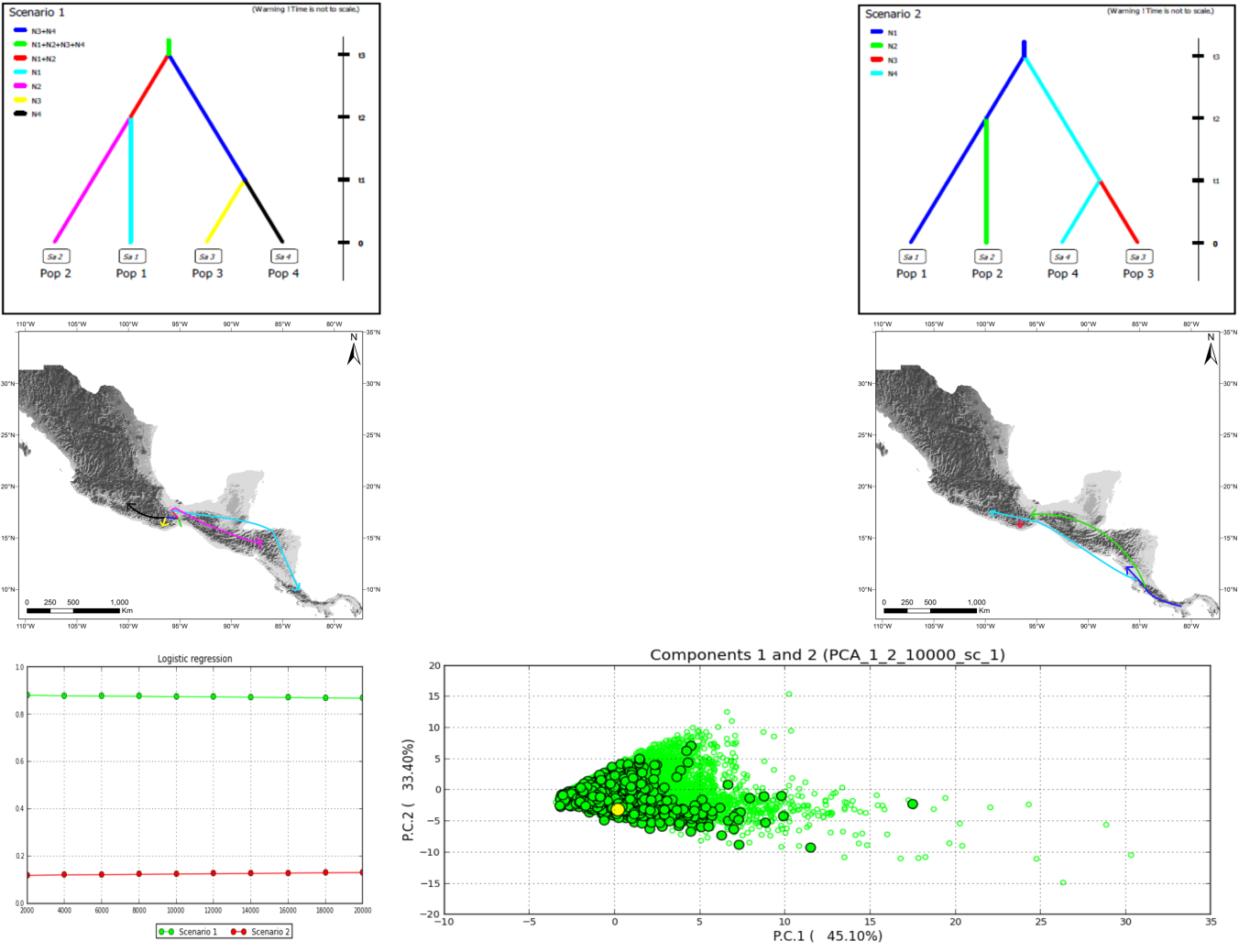


Fig. 11: Competing scenarios of *Eupherusa*, posterior probability of scenarios assessed with a logistic regression on the 1% of the simulated datasets closest to the observed data, and model checking for the best supported scenario (scenario 1) applying a PCA on test statistic vectors to visualize fit between simulated and observed datasets.
